# Effects of yoga on cardiometabolic risks and fetomaternal outcomes are associated with serum nitric oxide in gestational hypertension: a randomized control trial

**DOI:** 10.1038/s41598-022-15216-4

**Published:** 2022-07-12

**Authors:** Kuzhanthaivelu Karthiga, Gopal Krushna Pal, Papa Dasari, Nivedita Nanda, Subramanian Velkumary, Palanivel Chinnakali, Manoharan Renugasundari, K. T. Harichandrakumar

**Affiliations:** 1 Department of Physiology, Advanced Center for Yoga, JIPMER, Gorimedu, Puducherry India 605 006; 2 Department of Obstetrics and Gynecology, JIPMER, Gorimedu, Puducherry India; 3 Department of Biochemistry, JIPMER, Gorimedu, Puducherry India; 4 Department of Preventive and Social Medicine, JIPMER, Gorimedu, Puducherry India; 5 Department of Biostatistics, JIPMER, Gorimedu, Puducherry India

**Keywords:** Physiology, Health care, Medical research

## Abstract

Gestational hypertension (GH) is associated with adverse cardiometabolic and pregnancy outcomes. Though yoga is known to be beneficial in pregnancy, the effects of yoga rendered for twenty weeks starting from 16th week of gestation in pregnant women having risk of GH on the incidence of hypertension, cardiometabolic risks and fetomaternal outcomes have not been studied. A randomized control trial was conducted on 234 pregnant women having risk of GH receiving standard antenatal care (Control group, n = 113), and receiving standard care + yoga (Study group, n = 121). Interventions were given for twenty weeks starting at 16th week of gestation. Baroreflex sensitivity (BRS), heart rate variability (HRV), insulin resistance, lipid-risk factors, and markers of inflammation, oxidative stress and vascular endothelial dysfunction (VED) were assessed before and after intervention. Incidence of new-onset hypertension, level of cardiometabolic risks at 36th week, and fetomaternal-neonatal outcomes in the perinatal period, were noted. The link of hypertension, pregnancy outcomes and cardiometabolic risks with nitric oxide (NO), the marker of VED was assessed by analysis of covariance, Pearson’s correlations, and multilinear and logistic regressions. In study group, 6.61% women developed hypertension compared to 38.1% in the control group following 20-week intervention and there was significant decrease in risk of developing GH (RR, 2.65; CI 1.42–4.95). There was less-painful delivery, decreased duration of labor, increased neonatal birthweight and Apgar score in study group. Increase in total power of HRV (β = 0.187, *p* = 0.024), BRS (β = 0.305, *p* < 0.001), and decrease in interleukin-6 (β =  − 0.194, *p* = 0.022) had significant association with increased NO. Twenty weeks of practice of yoga during pregnancy decreases the incidence of hypertension, improves fetomaternal outcomes, and reduces cardiometabolic risks in pregnant women having risk of GH. Decreased blood pressure, increased HRV, BRS and birth weight and decreased inflammation were associated with improved endothelial function.

Trial registration: Clinical Trials Registry of India (CTRI), registration number: CTRI/2017/11/010608, on 23.11.2017.

## Introduction

Hypertensive disorders of pregnancy (HDP) that occurs in 7–10% of pregnancies worldwide include gestational hypertension, preeclampsia and preeclampsia superimposed on chronic hypertension^1^. Gestational hypertension (GH) is defined as the level of blood pressure (BP) of at least 140/90 mmHg or above measured on two separate occasions, more than 4 h apart, and arising de novo after the 20th week of pregnancy in a previously normotensive woman^1^. Women with GH are reported to be at increased risk of complications both antenatally and postnatally^2^. WHO has estimated the incidence of preeclampsia to be seven times higher in developing countries (2.8% of live births) than in developed countries (0.4%)^3^. In developing countries like India, about 50% of GH leads to preeclampsia, which may culminate in eclampsia if not treated early and effectively^4^. The maternal mortality in eclampsia in India is estimated to be 20–33%^5^. Inspite of qualitative antenatal care provided in secondary and tertiary care hospitals, the maternal morbidities and mortalities are quite high in preeclampsia and eclampsia^6,7^. Therefore, it is important to identify the women at high risk for GH, predict the development of GH and initiate the treatment early in pregnancy to prevent the occurrence of GH.

Currently, no pharmacological treatment is available to cure preeclampsia, although acetylsalicylic acid (aspirin) is commonly prescribed prophylactically in women at high risk of HDP^6^. The only known cure in HDP is to deliver the baby and remove the placenta. The pharmacological treatment of maternal hypertension with anti-hypertensive drugs is commonly reserved for reducing maternal symptomology including organ failure and stroke^7^, as pharmacological interventions have been limited due to their concerns about the side effects on fetal growth and maturation, and for the preference of pregnant women for alternative medicines^8^. Therefore, complementary health approaches such as mind–body meditation, massage therapy, relaxation techniques (breathing exercises, guided imagery, and progressive muscle relaxation), tai chi, qi gong, healing touch, hypnotherapy, acupuncture, chiropractic and osteopathic interventions have been tried in the treatment of HDP, though they have not been scientifically proved to be very effective^9^.

An integrated approach to yoga during pregnancy has been demonstrated to be safe and reported to improve birth weight, decrease preterm labor, and decrease intrauterine growth retardation (IUGR) either in isolation or associated with pregnancy-induced hypertension (PIH)^10^. A recent randomized controlled trial (RCT) of integrated yoga in HDP has reported decrease in BP and increase in maternal comfort^11^. We have reported the effectiveness of a slow pranayamic breathing exercise on reduction in the incidence of PIH^12^. Therefore, in the present study we have investigated the efficacy of a structured yoga module consisting mainly of basic asanas and slow pranayamas on the maternal-neonatal outcomes in pregnant women having risk of GH and we have assessed the plausible physiological and biochemical basis of improvements following yoga intervention.

In HDP, the maternal morbidity and mortality are mainly due to cardiovascular (CV) complications^13^. Though the exact mechanisms that contribute to the development of CV risks in GH have not been fully elucidated, sympathovagal imbalance attributed by oxidative stress and retrograde inflammation have been proposed to be the physiological basis of CV problems in PIH^14^. Inflammation has been reported to play a major role in the pathogenesis of preeclampsia^15^ and IL-6 has been identified as a biomarker of hypertension during pregnancy^16^.

We have reported decreased baroreflex sensitivity (BRS) as a physiological marker of CV risks in pregnancy-induced hypertension (PIH)^17^. Recently we have reported the association of decreased level of nitric oxide (NO) with decreased BRS in GH^18^. Recently we have also demonstrated the improvement in autonomic functions including BRS following 12 weeks practice of pranayama in prehypertension^19^. Yoga has proved to have some benefit in preventing cardiovascular diseases^20^. Inflammatory biomarkers including IL-6 are shown to be decreased by practice of yoga^21^. Yoga improves HRV, BRS and cardiometabolic profile in health and diseases primarily by improving vagal tone and decreasing sympathetic activity^22,23^. As such, GH is primarily a disorder of autonomic dysregulation^14^. Till date, there are no reports of yoga practiced for a longer duration during the course of pregnancy on the incidence of hypertension, cardiometabolic risks and maternal-neonatal outcomes in women having risk of GH. We hypothesized that twenty weeks practice of yoga starting from 16th week of gestation in women having risk of GH will result in improvement in their cardiovagal modulation and decrease in sympathetic tone, which will lower the incidence of hypertension, lessen the inflammatory milieu, decrease the cardiometabolic stress and improve the fetomaternal outcomes. Therefore, in the present study, we have assessed the role of 20-week yoga practice on the incidence of new-onset hypertension, cardiometabolic profile and maternal-neonatal outcomes and their association with NO in pregnant women with risks of GH.

## Methods

### Study design

This parallel-design single-blinded randomized control trial was conducted during January, 2018–December, 2020, in the Department of Physiology, Jawaharlal Institute of Postgraduate Medical Education and Research (JIPMER), Puducherry, India, after obtaining approvals of PhD Doctoral Committee, Scientific Advisory Committee and Institute Human Ethics Committees of JIPMER, Puducherry. All the methods were performed in accordance with the institutional guidelines and regulations. The trial was registered prospectively at Clinical Trials Registry of India (CTRI) with registration number: CTRI/2017/11/010608 (Registered on: 23/11/2017).

### Sample size calculation

Sample size was estimated with a minimum expected difference in the incidence of gestational hypertension between the pregnant women receiving yoga and standard care, and pregnant women receiving standard care alone is 15% at 5% level of significance and 80% power^12^. The estimated sample size is 152 in each group.

### Inclusion criteria

The normotensive pregnant women before 16 weeks of pregnancy and having any of the established risk factors for GH, such as family history of preeclampsia or GH, preeclampsia or GH in previous pregnancy, extremes of reproductive age, first pregnancy, multiple pregnancy, etc. Women having body mass index (BMI) > 35, diastolic blood pressure (DBP) < 90 mmHg, and systolic pressure (SBP) < 140 mmHg at the first antenatal check-up were recruited for the study.

### Exclusion criteria

The subjects with unknown last menstrual date, women not willing to deliver in JIPMER hospital, subjects undergoing any kind of yoga therapy and/or structured exercises, women known to have hypertension, cardiac arrhythmias, and endocrinal disorders including diabetes mellitus before pregnancy, on any medications and those who have received oral contraceptive pills within 6 months prior to pregnancy.

### Randomization and masking

**Figure 1:**
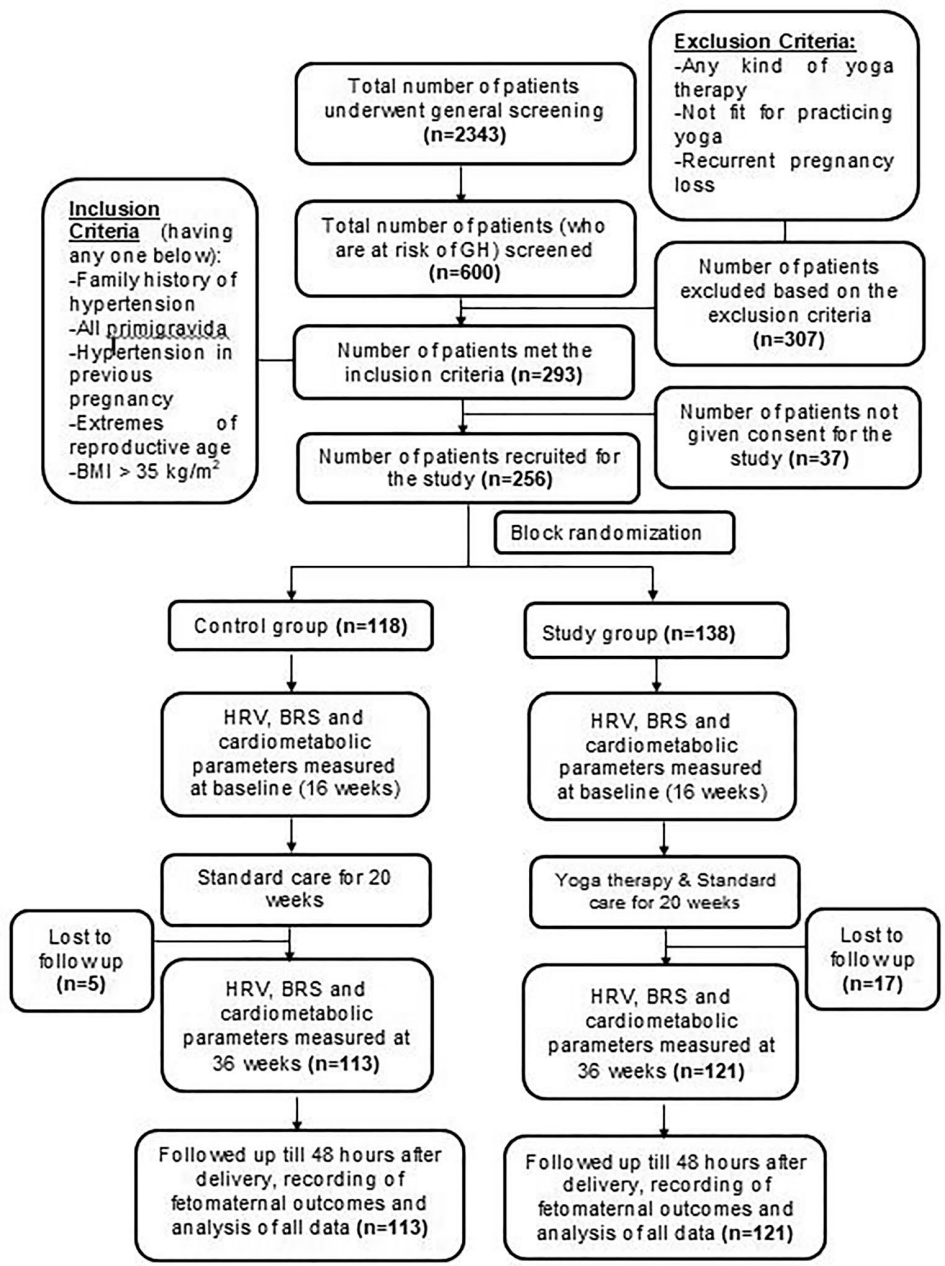
RCT consort chart.

A total of 2343 pregnant women with risk factors for GH at 16th week of pregnancy were screened in the obstetrics out-patient department (OPD) of JIPMER hospital. After scrutiny for inclusion and exclusion criteria, 256 subjects were recruited by the obstetrician and randomized into control group (n = 118) and study group (n = 138) by second author using block randomization (Fig. 1, RCT consort chart). For randomization, computer-generated random-number sequences were used. The random numbers were generated by the co-investigator from the Department of Preventive and Social Medicine. Block randomization with random block sizes of 4, 6 and 8 was followed. The sequences were concealed (allocation concealment) using serially numbered opaque sealed envelopes (SNOSE). The care-givers, participants and outcome-assessors were blinded. Five subjects from control group and 17 subjects from study group could not come to JIPMER hospital for their last-trimester visit due to COVID-19 lockdown. The total of 234 subjects (Control group, n = 113; Study group, n = 121) who attended antenatal check-ups in all the three trimesters and participated in all the investigations, were followed up till term.

### Brief procedure

Subjects reported to Autonomic Function Testing (AFT) and Cardiovascular Research laboratories of physiology department between 8 to 9 AM. Written informed consent was obtained from each participant. Height, body weight and body mass index (BMI) were recorded. After 10 min of supine rest on a couch, their heart rate (HR), SBP and DBP were recorded.

#### Recording of heart rate variability (HRV)

Recommendation of the Task Force on HRV^24^, and the standard methods as practiced earlier^18^, were followed for short-term HRV recording. Lead II ECG was acquired at a rate of 1000 samples/sec during supine rest using BIOPAC MP-150 data acquisition system (BIOPAC Inc., USA). The data were transported from BIOPAC to a windows-based PC having AcqKnowledge software version 3.8.2 (Biomedical signal analysis group, University of Kuopio, Finland). The frequency-domain indices of HRV measured were total power (TP), normalized low-frequency power (LFnu), normalized high-frequency (HFnu), and ratio of low-frequency to high-frequency power (LF-HF ratio). The time-domain indices measured were mean and standard deviation of RR intervals (SDNN), square root of the mean of the sum of the squares of differences between adjacent RR interval (RMSSD), adjacent RR interval differing more than 50 ms (NN50), and NN50 counts divided by all RR intervals (pNN50).

#### Blood pressure variability (BPV) measurement including BRS

Continuous BPV was measured using Finapres (Finometer version 1.22a, Finapres Medical Systems, Amsterdam, The Netherlands), a noninvasive continuous hemodynamic-monitor that works on the principle of volume-clamp technique. The detailed procedures of BPV measurement were followed, as described earlier^17^. A continuous BP recording was done for a period of 10 min. The reconstructed brachial pressure tachogram was acquired through a PC-based data acquisition system^25^. The recording of BPV parameters included heart rate, SBP, DBP, mean arterial pressure (MAP), rate-pressure product (RPP), interbeat interval, left ventricular ejection time (LVET), stroke volume (SV), cardiac output (CO), total peripheral resistance (TPR), and BRS.

#### Estimation of biochemical parameters

Five ml of fasting blood sample was collected, and serum was separated for estimation of all biochemical parameters following the procedures as adopted earlier^18^. Blood glucose was estimated by enzymatic method (Randox, India). Insulin was measured using ELISA kit (Calbiotech, USA). Homeostatic model assessment of insulin resistance (HOMA-IR) was calculated using the formula blood glucose (mg/dL) × insulin (µIU/mL)/405. HbA1c was determined from whole blood using commercial kits for turbidimetric immunoassay (Quantia, Tulip diagnostics). Lipid parameters such as total cholesterol (TC), triglyceride (TG), high-density lipoprotein-cholesterol (HDL-c), low-density lipoprotein-cholesterol (LDL-c) were estimated using reagent method (Randox, India). Very low-density lipoprotein-cholesterol (VLDL-c) was calculated using the Friedwald’s formula (TG/5). Various lipid-risk factors were assessed and atherogenic index of plasma (AIP) was calculated by log_10_ of TG/HDL-c. Malondialdehyde (MDA) was measured by colorimetric assay-kit (Elabscience, USA), and high-sensitive C-reactive protein (hsCRP) (Calbiotech, USA), and interleukin-6 (IL-6) (Diaclone, France) were measured using ELISA kit, according to manufacturer instructions. Nitric oxide derivatives (nitrate and nitrite) were estimated by colorimetric method using microplate reader (Elabscience, USA).

### Intervention schedule

**Figure 2:**
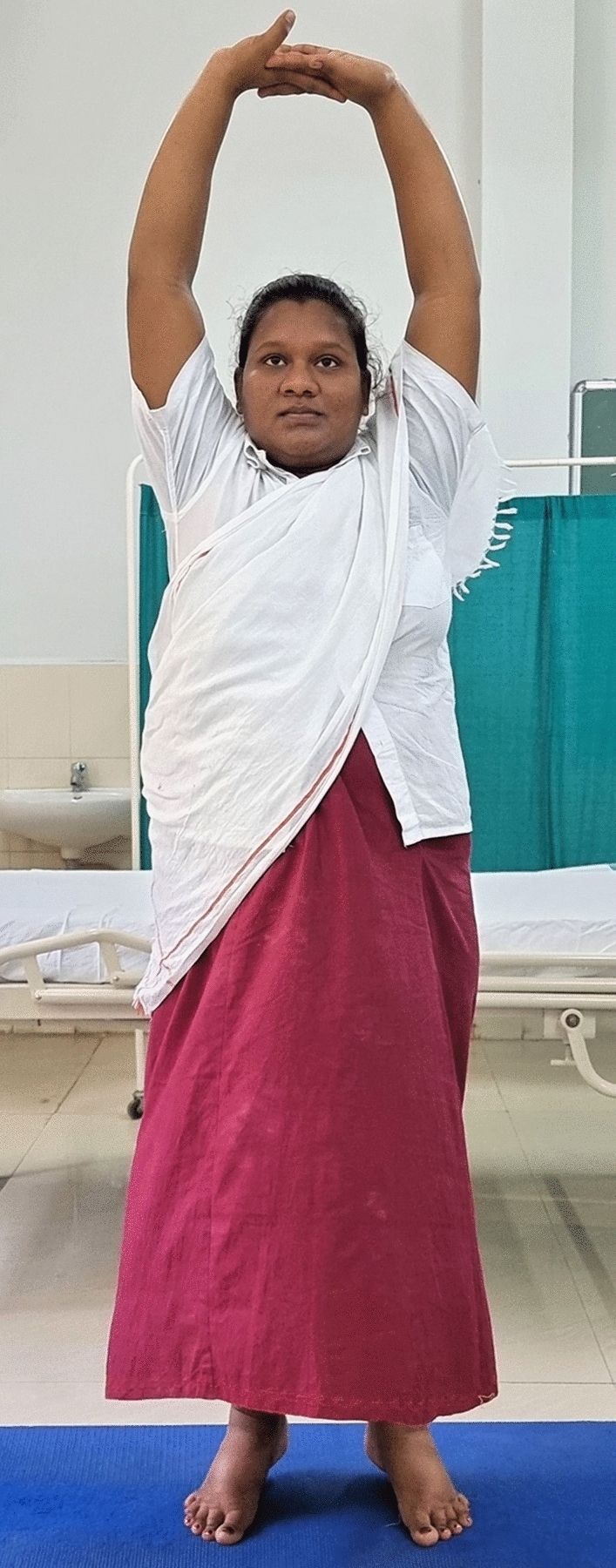
Tadasana.

**Figure 3:**
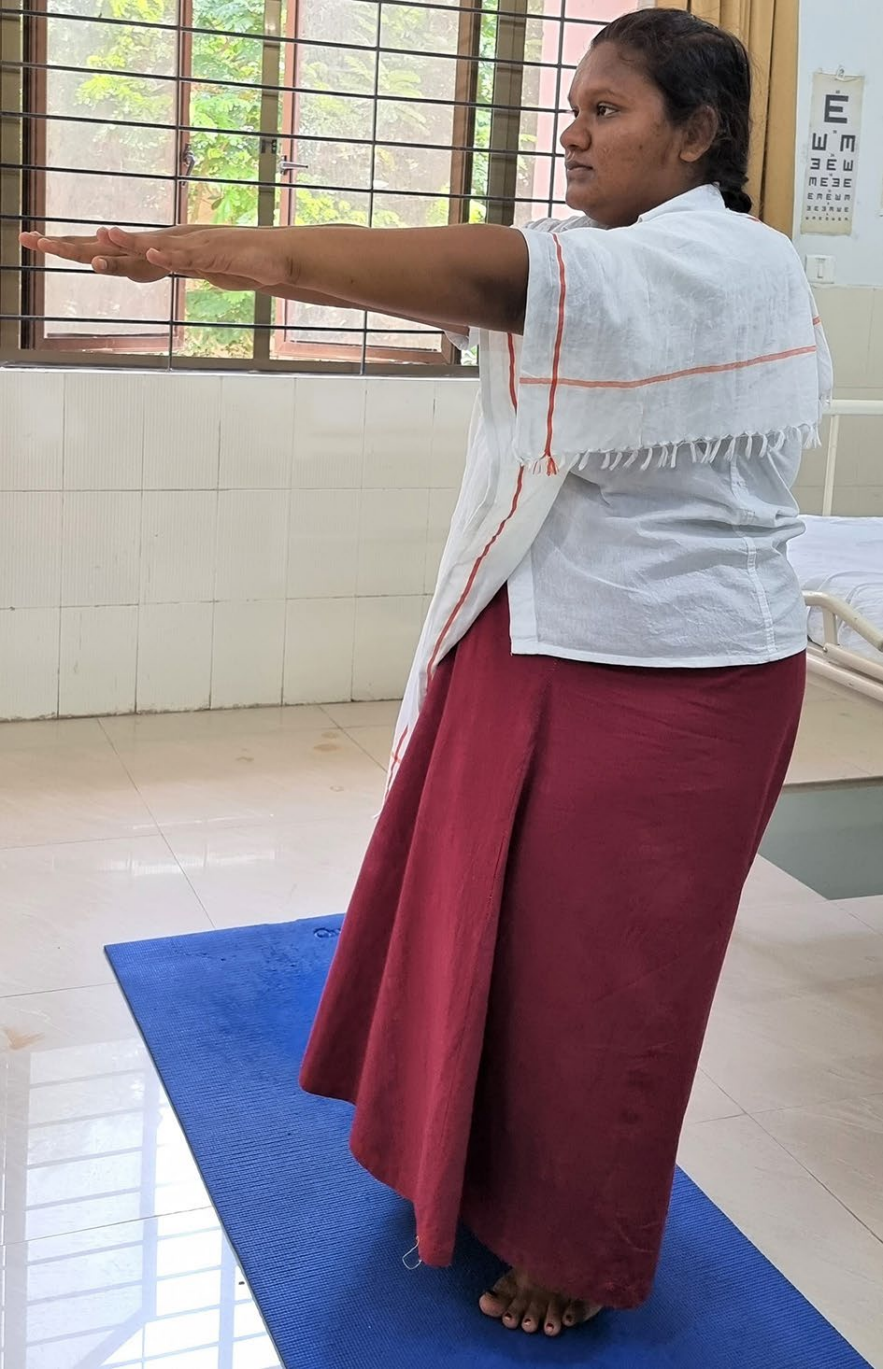
Utkatasana.

**Figure 4:**
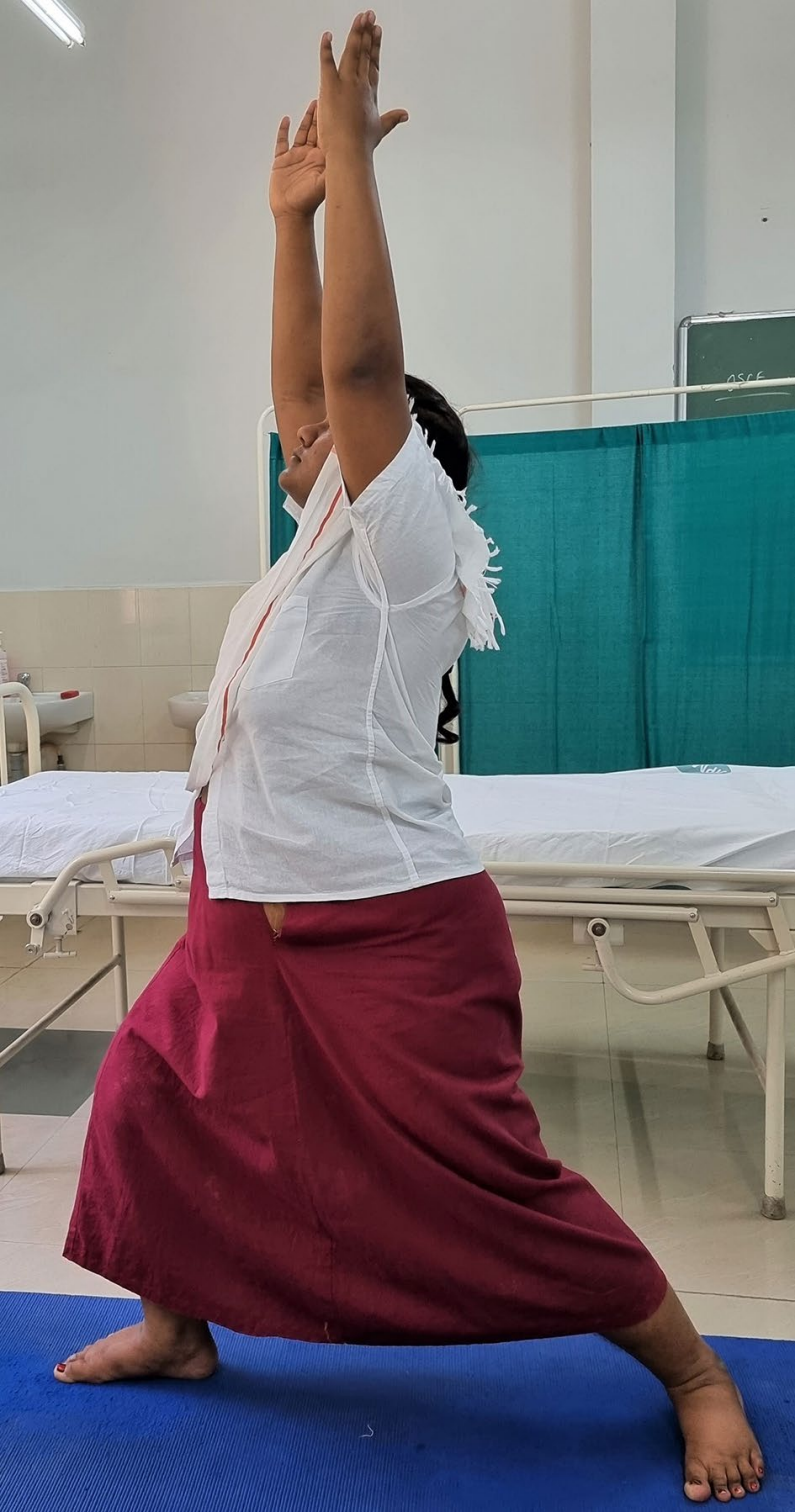
Virabhadrasana.

**Figure 5:**
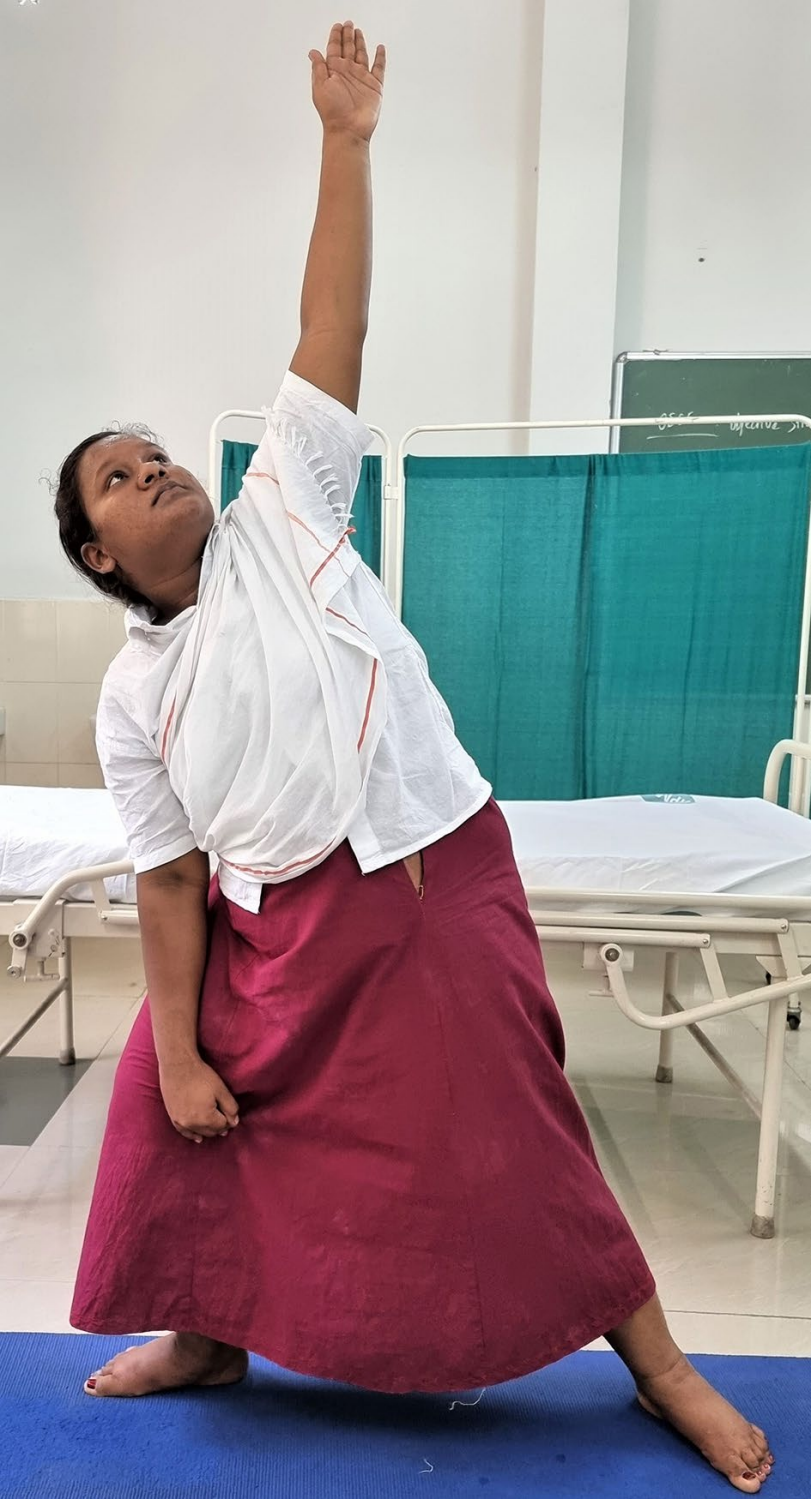
Trikonasana (Modified).

**Figure 6:**
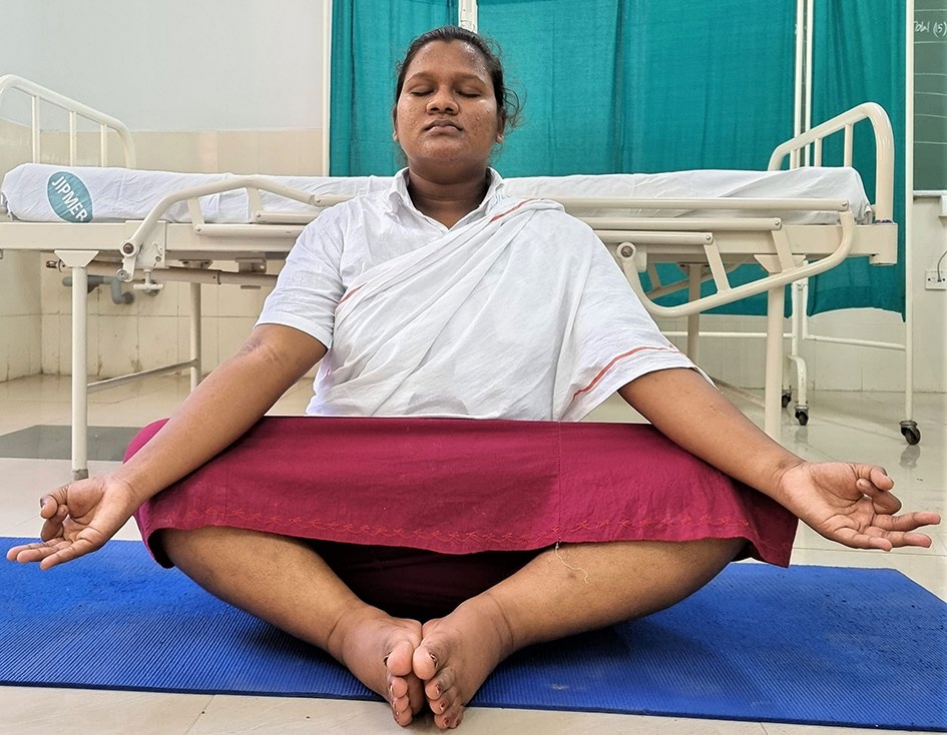
Titilasana.

**Figure 7:**
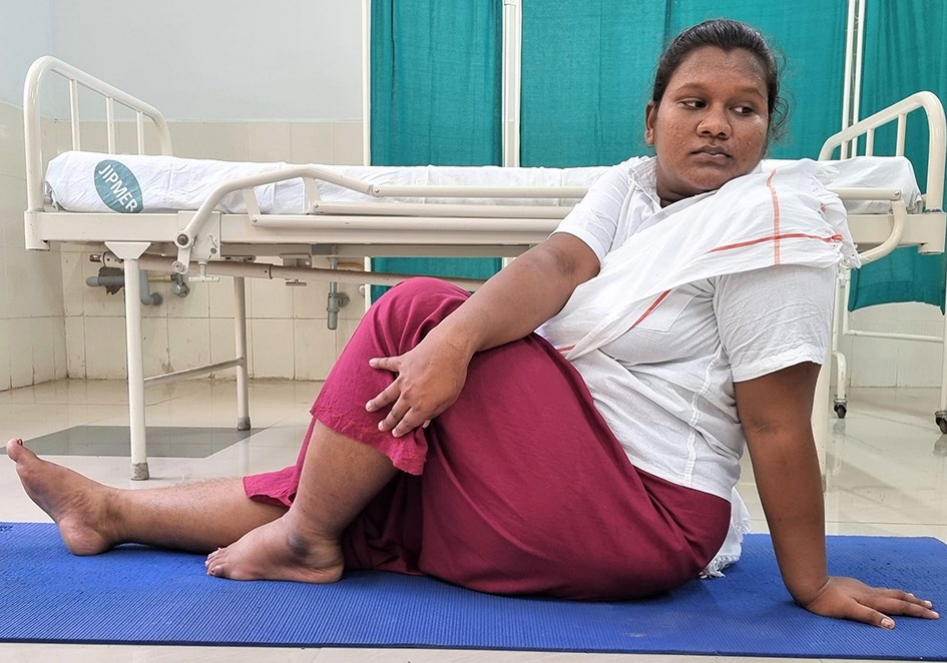
Vakrasana.

**Figure 8:**
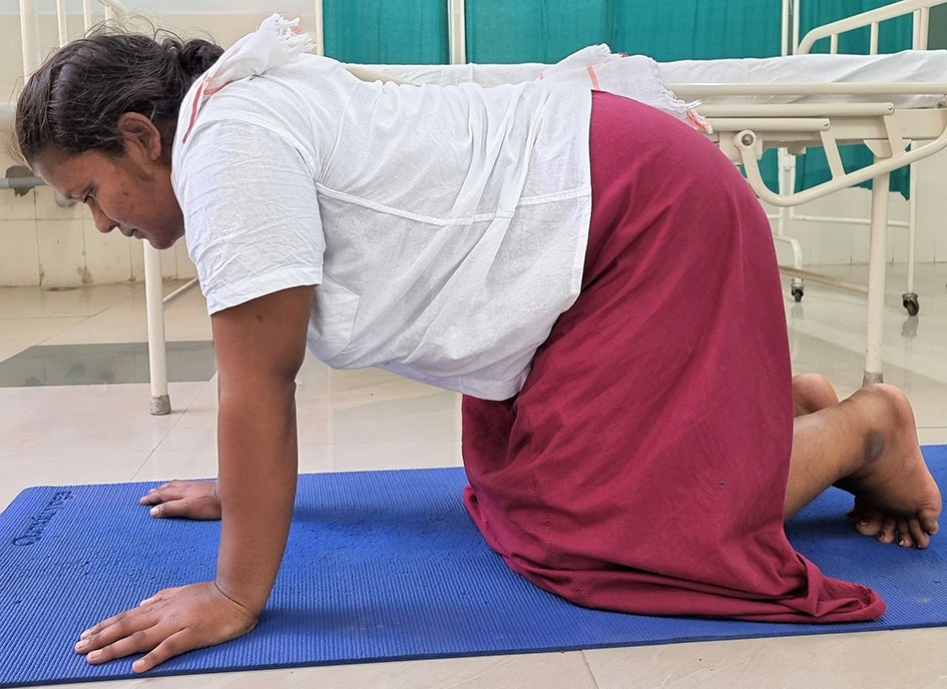
Marjariasana.

**Figure 9:**
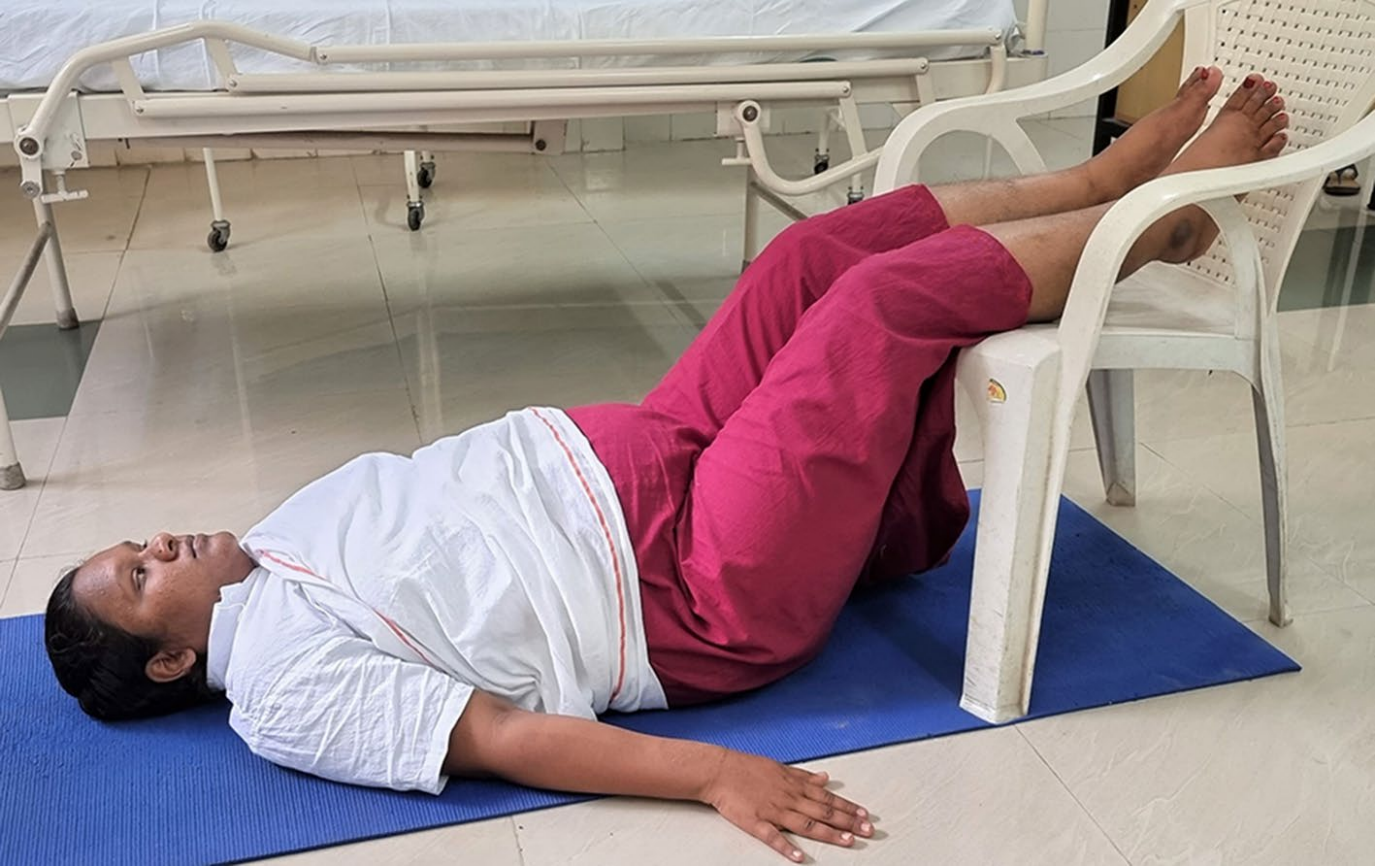
Uthita Padasana (modified).

**Figure 10:**
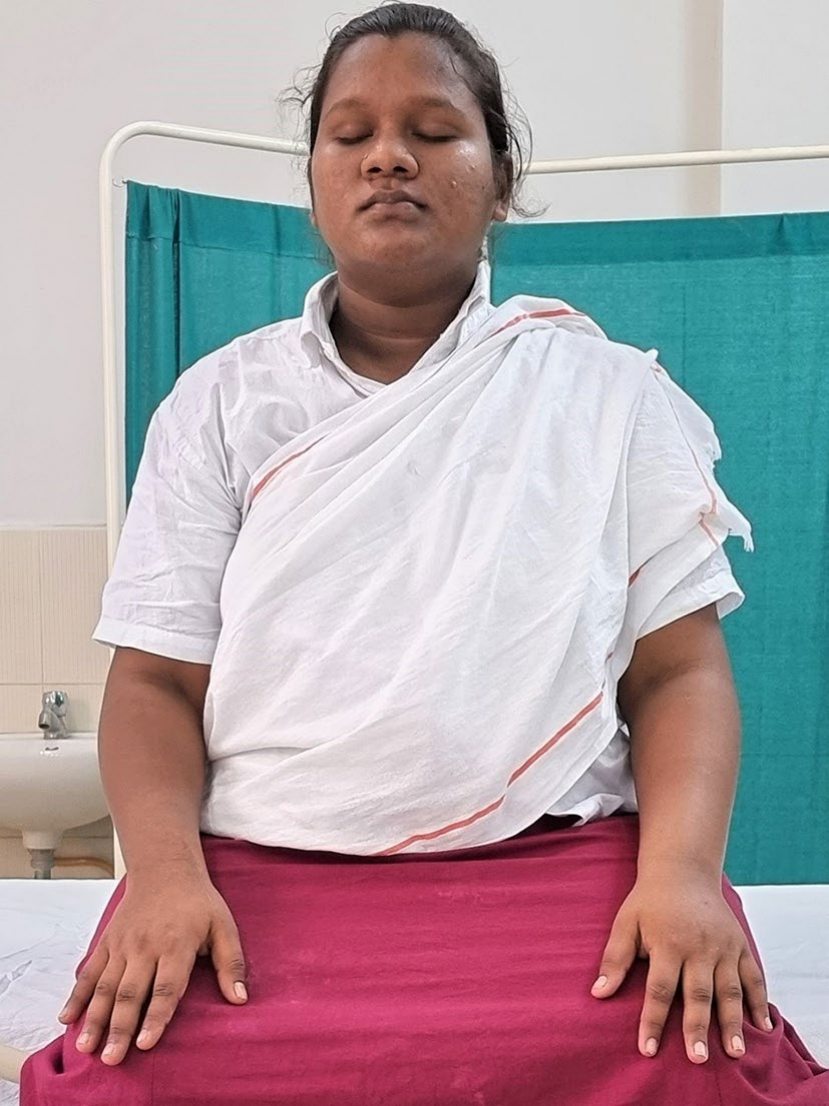
Sukhasana.

**Figure 11:**
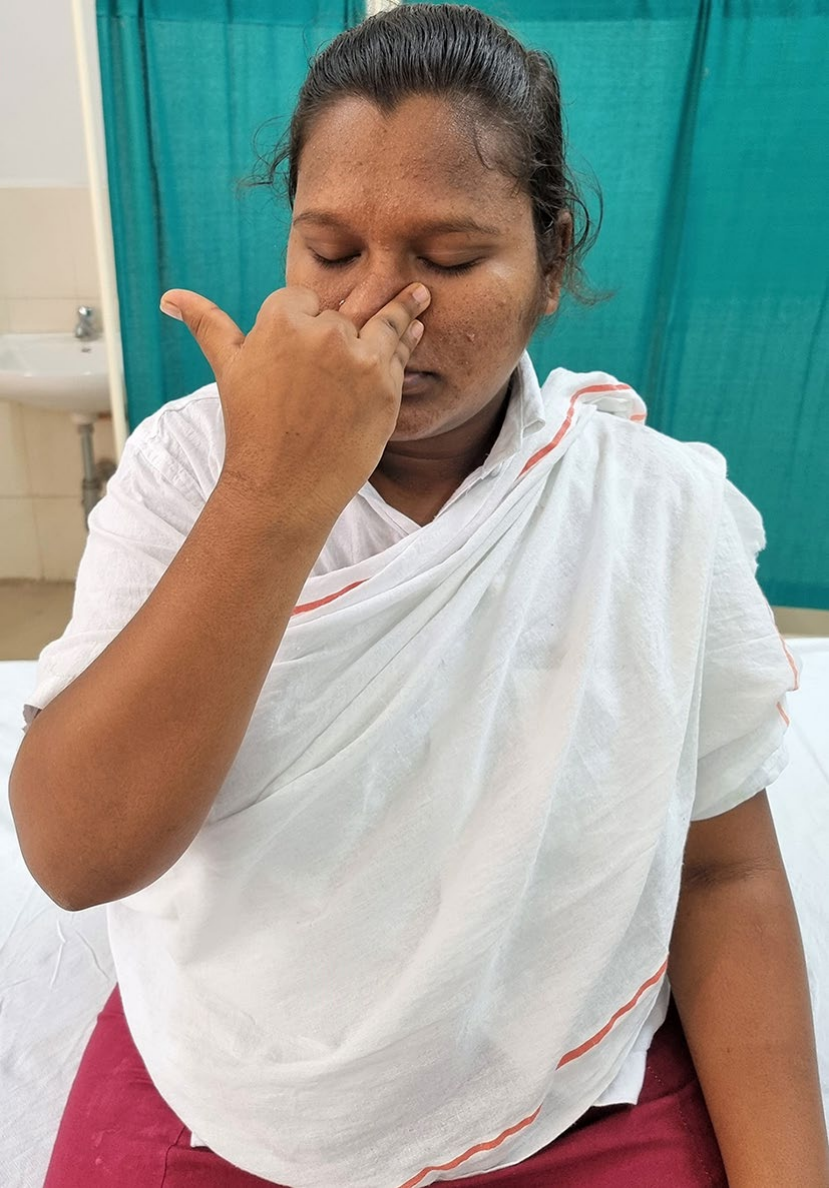
Anulom-Vilom or Nadisodhana pranayama.

**Figure 12:**
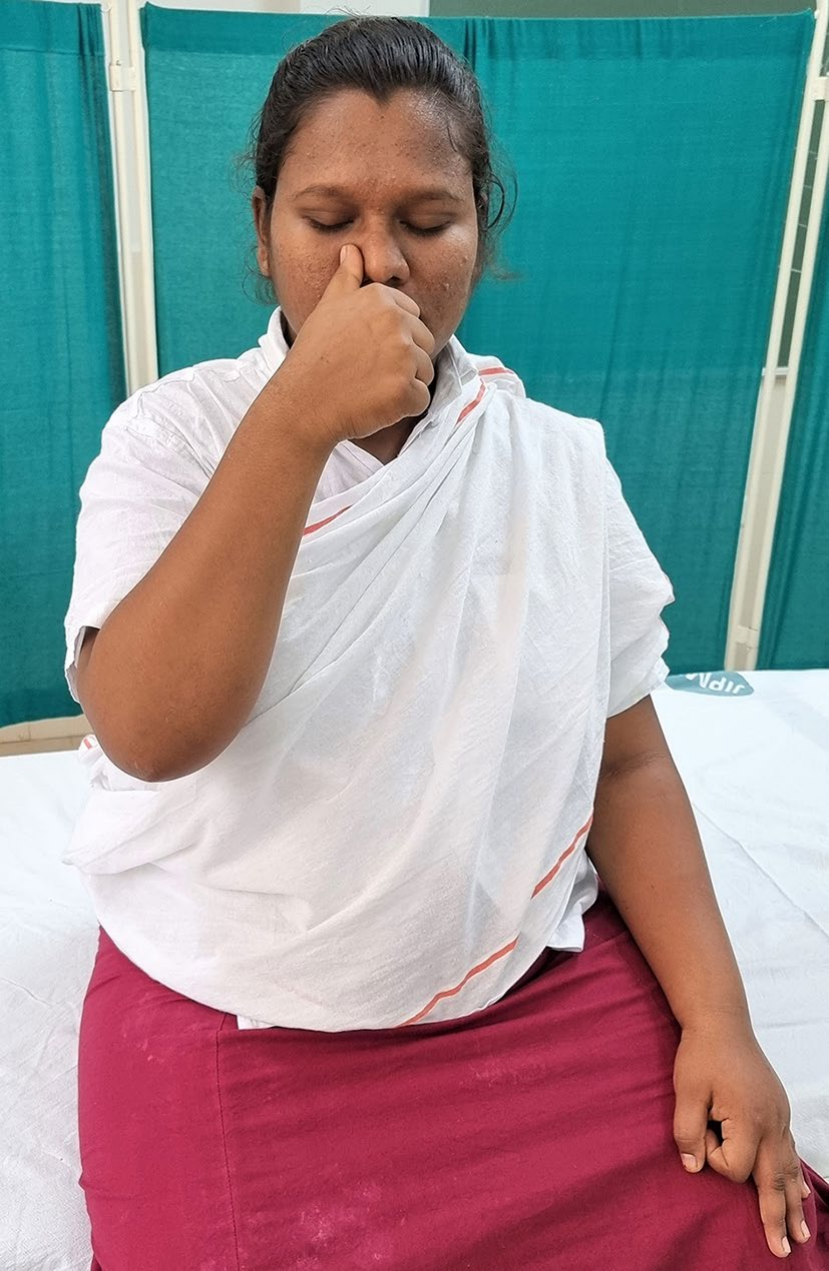
Chandranadi or left-nostril pranayama.

**Figure 13:**
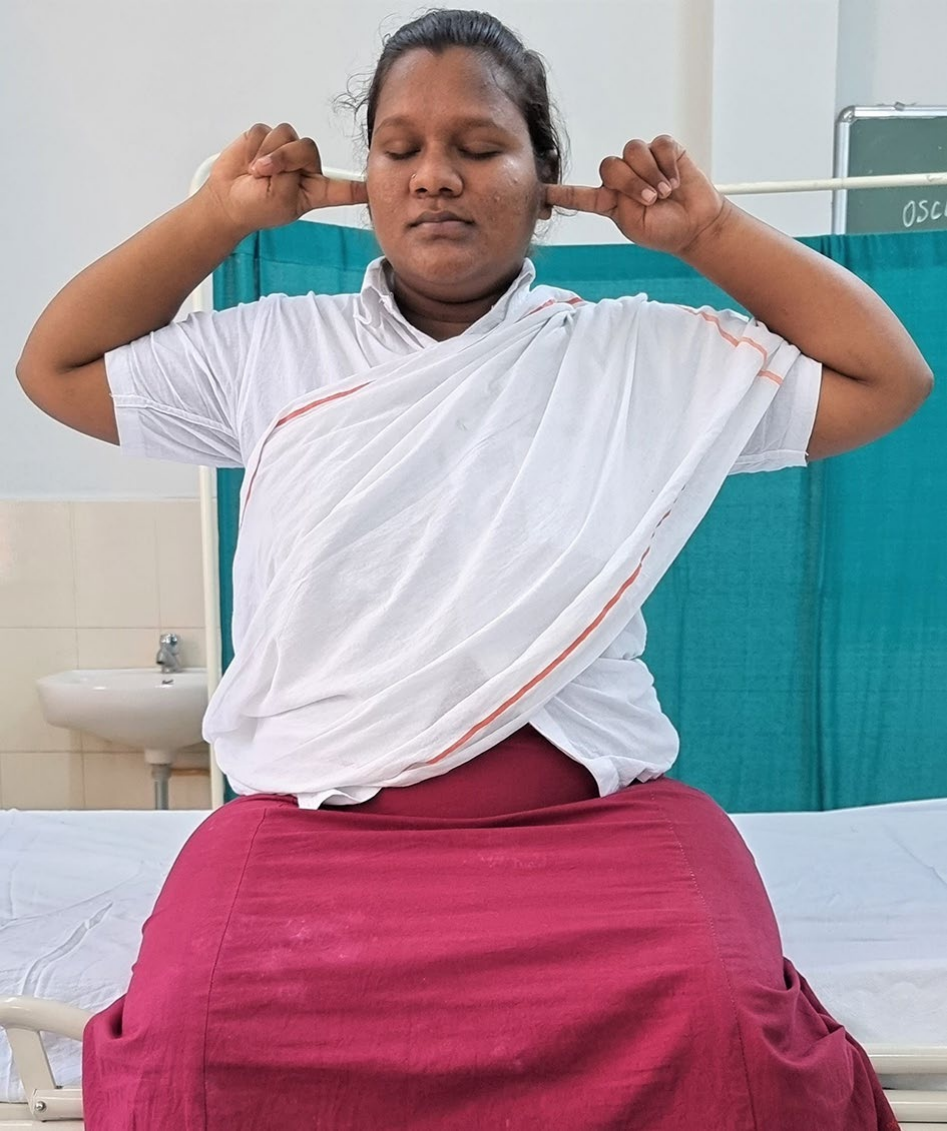
Bhramari or humming-bee pranayama.

**Figure 14:**
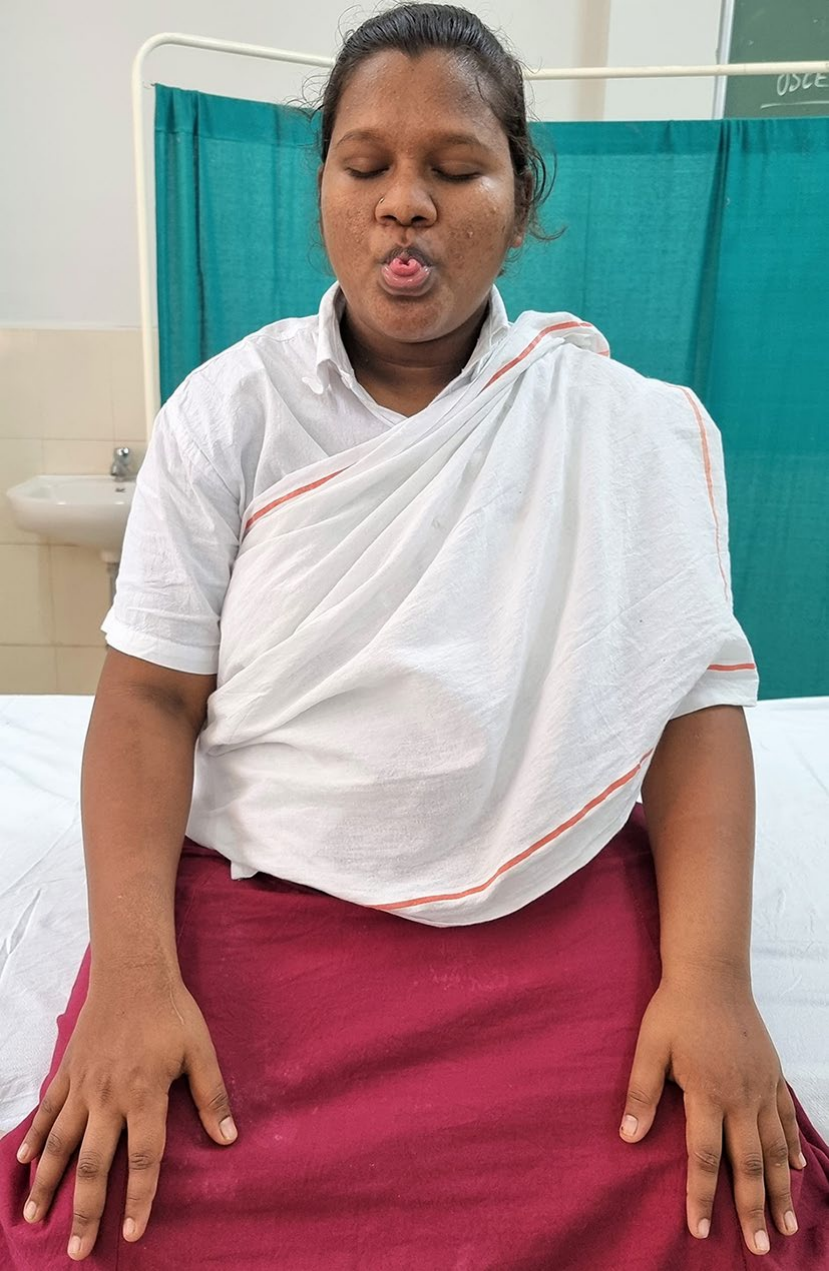
Sheetali or cooling pranayama.

**Figure 15:**
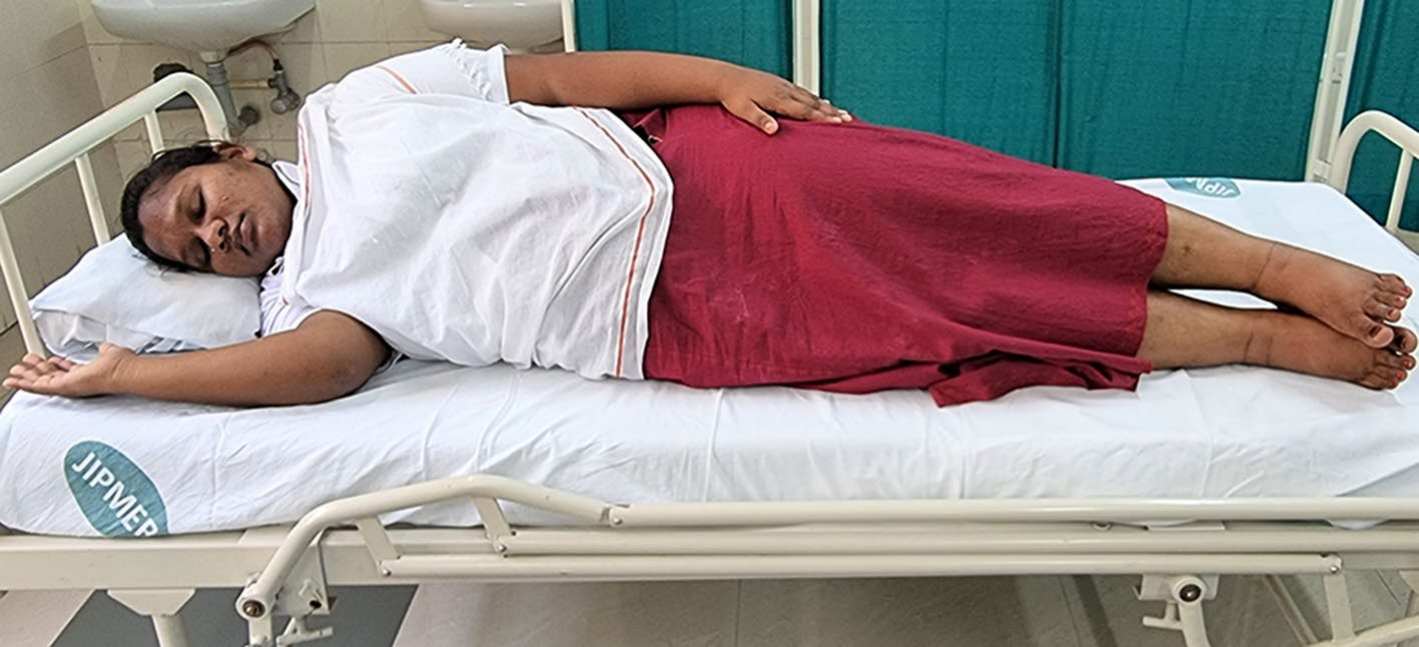
Relaxation in lateral posture.

The control group subjects were given standard antenatal care as routinely practiced for pregnant women at risk of HDP, in JIPMER hospital (Table 1) and they were not allowed to practice yoga. The study group participants were given a structured module of yoga therapy for twenty weeks (Table 2, Figs. 2, 3, 4, 5, 6, 7, 8, 9, 10, 11, 12, 13, 14, 15), in addition to standard antenatal treatment. The yoga module selected for the study is known to facilitate delivery and to reduce morbidity and mortality in high-risk pregnancies^26^. The participants were trained to perform yoga (Table 2) by the qualified yoga instructor in the Advanced Center for Yoga Therapy Education and Research (ACYTER), JIPMER. Initial five sessions were conducted to ensure thorough learning of yoga techniques by the participants, and subsequently they practiced yoga at home twice daily (between 6 to 7 am, and 6 to 7 pm). Each participant was provided with a diary and a calendar chart to make entries after each session of yoga. The participants were frequently contacted through their phone, WhatsApp and email to assess the regularity of practice. The family members were advised to motivate and supervise the yoga practice by the participant. The family members were also contacted frequently to ensure that the participants are doing yoga regularly and they were advised to send few pictures or short video-clips of yoga sessions recorded randomly on different days. Supervised sessions in the hospital premises were conducted during their regular monthly antenatal checkup. The practice of yoga protocol was followed till 36th week of gestation starting from 16th week. However, participants were advised to continue yoga practice till delivery.

**Table 1:** Treatment received by pregnant women in control group and study group.

S. no.	Control group	Study group
1	Tab. **Aspirin**: 75 mg after lunch, starting from 12th week and continued upto term. Stopped before planned labor or preterm labor or if there is any bleeding or intrauterine death	Tab. **Aspirin**: 75 mg after lunch, starting from 12th week and continued upto term. Stopped before planned labor or preterm labor or if there is any bleeding or intrauterine death
2	(i) Tab. Folate—5 mg, once daily (ii) FST (Ferrous Sulphate Tablet)—200 mg, twice daily (iii)Tab. Calcium—500 mg, once daily (iv)Tab. Vitamin C—100 mg, once daily	(i)Tab. Folate—5 mg, once daily ii) FST—200 mg, twice daily iii) Tab. Calcium—500 mg, once daily iv) Tab. Vitamin C—100 mg, once daily
3	**For those who had SBP 140 mmHg or above and/or DBP 90 mmHg or above** (In control group, 43 subjects had SBP 140 mmHg or more and 14 among these 43 subjects had DBP 90 mmHg or above): Tab. Labetalol 100 mg TID (thrice daily) **or** Tab. Nifedipine 10 mg TID (thrice daily)	**For those who had SBP 140 mmHg or above and/or DBP 90 mmHg or above** (In study group, 8 subjects had SBP 140 mmHg or more and none of them had DBP 90 mmHg or above): Tab. Labetalol 100 mg BD (twice daily) **or** Tab. Nifedipine 10 mg BD (twice daily)
4	Routine antenatal counseling	Antenatal counseling and yoga therapy (Details of yoga intervention are given in Table 2)

**Table 2:** The schedule of yoga intervention.

S. no.	Type of yoga techniques practiced		Duration
1	Sukshma vyayama and Asanas	Sukshma vyayama: Simple warm up shoulder exercises	1 min
		Tadasana: The subject stands on the toe with both the hands hooked and outstretched above the head (Fig. 2)	30 s
		Utkatasana: The subject extends both the forelimbs forward parallel to the ground keeping the trunk, neck and head straight, and comfortably bends down at the knee joint (Fig. 3)	30 s
		Virabhadrasana: The subject stands straight with both the legs placed widely apart in front and back and keeps the upper limbs stretched vertically upward above the head, and then bends down comfortably at knee joint (Fig. 4)	30 s
		Trikonasana (Modified): From standing posture, the subject assumes the shape of a triangle (trikona) by bending the trunk to one side keeping one hand straight upward (and looks upward) and supports the body by resting the other hand on the bent-thigh (Fig. 5)	30 s
		Titilasana (The subject sits comfortably by apposing the sole of both the feet, and keeps both the hands straight in padmasana mudra. Keeps the trunk of the body, head and neck straight) (Fig. 6)	30 s
		Vakrasana: The subject sits comfortably with one leg straight and other leg bent at knee and twists the body to the side, supports the body with one hand placed on the floor and the other hand kept on the bent-thigh (Fig. 7)	30 s
		Marjariasana: The subject assumes the posture of a cat (marjari) by kneeling down and resting the body with the help of stretched forelimb rested on the ground. Trunk, head and neck kept parallel to the ground (Fig. 8)	30 s
		Uthita Padasana (modified): In the lying supine posture with both the hands kept on the side of the body, the subject lifts the leg gently above the ground and places the leg on a chair or stool (Fig. 9)	30 s
		Sukhasana: The subject sits comfortably on the side of the bed or on a chair keeping the forelimbs on the thigh and trunk of the body straight (Fig. 10)	30 s
2	Slow Pranayamas	Anulom-Vilom or Nadisodhana pranayama (also called alternate-nostril breathing). This is a slow and deep breathing performed through alternate nostril. The subject breathes in left nostril while the right nostril is closed, and then breathes through right nostril with the left nostril closed. Thus, breathing is repeated in alternate nostrils (Fig. 11)	5 min
		Chandranadi or left-nostril pranayama (Left nostril breathing): The subject performs slow and deep breathing through left nostril with right nostril closed (Fig. 12)	5 min
		Bhramari or humming-bee pranayama: The subject takes deep inspiration followed by slow and prolonged expiration. During expiration, humming sound is produced with external meatus of both ears plugged by index fingers, and mouth kept closed (Fig. 13)	5 min
		Sheetali or cooling pranayama: The subject takes deep inhalation through mouth with tongue folded into a groove that allows cool air to enter, and then slowly exhales through the nostrils with mouth closed (Fig. 14)	5 min
3	Shavasana (modified)	Relaxing the body after lying comfortably in lateral posture (Fig. 15)	5 min
Total duration			30 min

Pictures in the Figs. 2, 3, 4, 5, 6, 7, 8, 9, 10, 11, 12, 13, 14 and 15 are photographs of a pregnant woman in the study group during her practice of different yoga techniques, at 34th week of pregnancy.

All the parameters were recorded in both study group and control group twice: once before intervention (Pre-Test) at 16th week, and the next after 20-week intervention (Post-Test) at 36th week. The participants of both groups were followed-up till 48 h of delivery to note the incidence of development of hypertension, and to assess the fetomaternal-neonatal outcomes.

As adverse events have been reported to be associated with yoga^27^, participants were regularly contacted and monitored for development of any harmful or undesirable symptoms during the intervention period.

### Assessment of fetomaternal and neonatal outcomes

Maternal outcomes were measured in terms of new-onset of hypertensive disorders of pregnancy (GH, preeclampsia and eclampsia). The number of patients who developed hypertension in both the groups and received antihypertensive medications was noted. Premature delivery, intrauterine growth retardation (IUGR), premature rupture of membrane (PROM), mode of delivery and duration of labor were noted from the obstetric data. Comfort during labor and grading of labor pain were assessed using numeric pain rating and pain mastery scale^28^, by interviewing the women. Neonatal outcomes were measured in terms of birth weight, ‘Appearance, Pulse, Grimace, Activity, and Respiration’ (APGAR) score, respiratory distress syndrome (RDS) and neonatal intensive care unit (NICU) admission.

### Outcomes

The primary outcome was to assess the incidence of new-onset hypertension following twenty weeks of intervention. The other outcomes were to assess the impact of interventions on cardiometabolic risks, pregnancy outcomes, and fetomaternal outcomes and to assess the link of cardiometabolic risks (insulin resistance, lipid risk factors, oxidative stress, inflammation) and fetomaternal outcomes to alteration in endothelial function (serum level of NO) following intervention.

### Statistical analysis

Statistical Package for the Social Sciences (SPSS) version 13 (SPSS Software Inc., Chicago, IL, USA) was used for statistical analysis. All data were expressed as mean ± SD and median (interquartile range). Normality of data was tested by Kolmogorov–Smirnov test.

The data of 22 dropouts (17 in study group and 5 in control group) were addressed as per the standard practice of handling missing data in RCT^29^. As per Little’s MCAR (missing completely at random) test, the dropout data were found to be missing completely at random. Therefore, observed data were used for analysis.

Comparison of post-intervention data between the control group and study group was done by analysis of covariance (ANCOVA). Chi square test, relative risk (Odd’s ratio) assessment and logistic regression were done for fetomaternal and neonatal outcomes. The strength of correlation of NO with cardiometabolic risk parameters was assessed by Pearson’s correlation.

The independent association of NO with various parameters such as MAP, BRS, TP, HOMA-IR, IL-6, MDA, AIP and birth weight was assessed by multiple regression analysis. Logistic regression was done for assessing independent association of categorical variables with NO. The P value < 0.05 was considered statistically significant.

### Ethics approval and consent to participate

Approval was obtained from Institute Ethics Committee (Human studies) of JIPMER (Reference number: JIP/IEC/2017/0271). Individual written informed consent was obtained from the participants before recruiting into the study.

### Consent for publication

All signed consent forms from the participants/patients have been obtained and preserved with me.

## Results

During the intervention period, three participants in the control group and two participants in the study group had common cold with mild fever for two days. They were treated with paracetamol tablets (500 mg, twice orally daily) and warm saline gargling of the throat. The patients in the study group who had common cold were advised not to practice yoga during the two days of illness. As the illness was comparable in both the groups, it appears that the ailment was not related to yoga. In general, the interventions in both the groups were found to be safe. The diary of yoga practice and all entries in the calendars were found to be in order.

### Fetomaternal and neonatal outcomes

Sample size was estimated to be 304 (152 in each group). However, we were able to recruit and randomize only 234 pregnant women with 113 in control group (receiving standard treatment alone) and 121 in study group (receiving yoga with standard treatment). The post power analysis for the comparison of the incidence of hypertension between the pregnant women who received yoga therapy along with standard care with the pregnant women received standard care alone, and power for this analysis was found to be 99%.

Out of 113 subjects in control group, 29 subjects (25.66%) developed GH, 11 subjects (9.73%) developed preeclampsia and 3 subjects (2.65%) developed eclampsia (Table 3). Out of 121 subjects in study group, 8 subjects (6.61%) developed GH and none of them had preeclampsia and eclampsia. GH was diagnosed when SBP ≥ 140 mmHg and/or DBP ≥ 90 mmHg in a woman who had normal blood pressure prior to 20th week of gestation and had no proteinuria (no excess protein in the urine). Preeclampsia was diagnosed when a woman with GH had increased protein in her urine (≥ 0.3 g protein in a 24-h urine specimen) and eclampsia was diagnosed when it was associated with new-onset, generalized tonic–clonic seizures. Forty-three subjects in the control group and eight subjects in the study group were treated with antihypertensive medications. The reduction in risk of developing GH among the subjects of study group compared to control group was quite significant (RR 2.65; CI 1.42–4.95; *p* < 0.001).

**Table 3:** Comparison of maternal and neonatal outcomes, and assessment of relative risks (RR) in women with risk of developing gestational hypertension in Control group and Study group in the perinatal period.

Parameters	Control group (n = 113)	Study group (n = 121)	Relative risk (95% CI)	P value
	Number (%)	Number (%)		
**Maternal outcomes**				
Developed HDP	43 (38.1)	8 (6.61)	3.93 (2.06–7.51)	0.001
i. Developed GH 29 (25.66)	29 (25.66)	8 (6.61)	2.65 (1.42–4.95)	< 0.001
ii. Developed Preeclampsia	11 (9.73)	Nil (0)	0.45 (0.39–0.52)	< 0.001
iii. Developed Eclampsia	3 (2.65)	Nil (0)	0.47 (0.41–0.54)	0.071
**Premature delivery**				
(< 36 weeks of gestation)	3 (2.65)	Nil (0)	0.47 (0.41–0.54)	0.071
**Pregnancy complications**				
IUGR	7 (6.19)	1 (0.82)	4.24 (0.67–26.67)	0.024
PROM	3 (2.65)	Nil (0)	0.47 (0.41–0.54)	0.071
Other complications	4 (3.53)	Nil (0)	0.47 (0.41–0.54)	0.037
**Mode of delivery**				
SVD	85 (75.22)	117(96.69)	0.21(0.08–0.54)	< 0.001
LSCS	25 (22.12)	4 (3.31)	4.13 (1.65–10.35)	< 0.001
Instrumental delivery	3 (2.65)	Nil (0)	0.47 (0.41– 0.545)	0.071
Duration of labor^*^				
1st stage labor	653.98 ± 128.48	401.48 ± 129.43		< 0.001
2nd stage labor	45.48 ± 13.04	39.29 ± 12.13		0.002
Total duration of labor	699.46 ± 127.77	440.78 ± 129.27		<0.001
**Comfort during labor**				
^†^By Numeric Pain Rating Scale^*^	6.69 ± 3.68	5.35 ± 1.44		0.003
^†^By Pain Mastery Scale				
(i) No Pain	Nil (0)	Nil (0)		
(ii) Manageable pain	3 (2.9)	99 (97.1)	0.17 (0.11–0.25)	<0.001
(iii) Bearable pain	31 (63.3)	18 (36.7)	1.51 (1.02–2.23)	0.018
(iv) Unbearable pain	54 (32.8)	Nil (0)	0.32 (0.26–0.40)	<0.001
**Neonatal Outcomes**				
Birth weight (kg)*	2.76 ± 0.34	2.91 ± 0.35		0.001
< 2.5 kg	14 (12.38)	4 (3.30)	0.41 (0.17–0.98)	0.009
2.5–3.4 kg	99 (87.61)	117 (96.70)	0.41 (0.17–0.98)	0.009
≥ 3.5 kg	Nil (0)	Nil (0)		
**APGAR score at 1st min**				
<7	12 (10.61)	2 (1.76)	3.78 (1.04–13.73)	0.004
≥ 7	101(89.38)	119 (98.34)	3.78 (1.04–13.73)	0.004
**APGAR score at 5th min**				
≥ 7	113 (100)	121 (100)		
RDS	3 (2.65)	Nil (0)	0.47 (0.41–0.54)	0.071
NICU admission	5 (4.42)	Nil (0)	0.47 (0.41–0.54)	0.019

The data are presented as number and percentage. ^*^ The duration of labor, assessment of comfort during labor by NPRS (Numeric Pain Rating Scale), and birth weight are expressed in Mean ± SD and comparison of these continuous data was done by independent *t* test.

P value < 0.05 was considered statistically significant.

HDP hypertensive disorders of pregnancy, GH gestational hypertension, IUGR intrauterine growth retardation, PROM premature rupture of membrane, SVD spontaneous vaginal delivery, LSCS lower segment caesarean section, APGAR appearance, pulse rate, grimace, activity and respiration, RDS respiratory distress syndrome, NICU neonatal intensive care unit.

^†^Numeric pain rating scale ranges from 0 to 10. The scale 0 represents “no pain”, 1 to 3 represents “mild pain”, 4 to 6 represents “moderate pain” and 7 to 10 represents “severe pain”. Pain mastery scale has 4 grading of pain: no pain, manageable pain, bearable pain and unbearable pain.

There was no premature delivery, no PROM, no complications, and 1 case of IUGR in study group subjects (Table 3). The LSCS was 3.31% in study group compared to 22.12% in control group. The reduction in risk of undergoing LSCS among the subjects of study group compared to control group was highly significant (RR, 4.13; CI 1.65–10.35; *p* < 0.001). The duration of labor and labor pain intensity were significantly reduced in study group.

There was significant improvement in birth weight (*p* = 0.001) and APGAR score (RR, 3.78; CI 1.04–13.73; *p* = 0.004) and there was no incidence of RDS and admission to NICU in study group.

### Physiological (HRV and BRS) parameters

**Figure 16:**
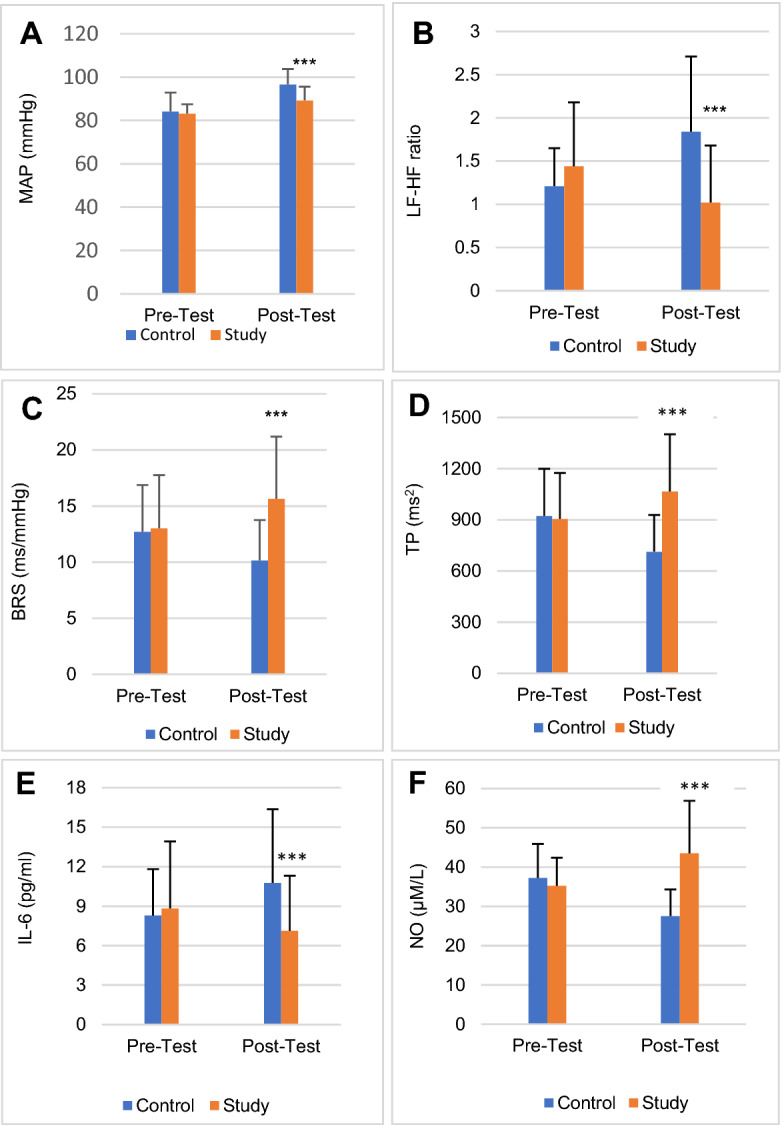
Comparison of MAP **(A)**, LF-HF ratio **(B)**, BRS **(C)**, TP **(D)**, IL-6 **(E)** and NO **(F)** in control and study groups before and after intervention. Results are shown as Mean ± SD. There was no significant difference in any of the parameters between control and study groups before yoga intervention (Pre-Test). Following intervention (Post-Test), MAP, LF-HF ratio and IL-6 were significantly decreased and BRS, TP and NO were significantly increased in the study group compared to the control group. ***p < 0.001.

BPV indices showed significant decrease (*p* < 0.001) in BHR, SBP, DBP, MAP (Fig. 16A), RPP, LVET, TPR and significant increase (*p* < 0.001) in BRS (Fig. 16C) in study group compared to control group following 20-week intervention (Table 4). The condition effect was quite high for BHR (F = 156.170, *p* < 0.001, η^2^ = 0.403), RPP (F = 160.439, *p* < 0.001, η^2^ = 0.410) and BRS (F = 79.217, *p* < 0.001, η^2^ = 0.255).

**Table 4:** Descriptive statistics and ANCOVA results of Post-Test (after 20 weeks of intervention) anthropometric, heart rate, blood pressure, HRV and BPV parameters between the control (pregnant women with risks of GH receiving routine standard antenatal care alone) and study group (pregnant women with risks of GH receiving routine standard antenatal care + Yoga) subjects.

Parameters	Pre-test	Post-test		Condition effect		
	Mean ± SD	Mean ± SD	Adjusted mean	F	P	Partial eta^2^
**Age (years)**						
Control	23.61 ± 3.74					
Study	24.16 ± 3.79					
**Body weight (kg)**						
Control	52.66 ± 8.44	60.35 ± 8.40	60.61			
Study	53.21 ± 9.45	60.59 ± 9.33	60.34	0.472	0.493	0.002
**BMI (kg/m**^**2**^**)**						
Control	21.88 ± 3.43	24.91 ± 3.43	25.03			
Study	22.14 ± 3.69	25.11 ± 3.52	24.99	0.049	0.825	0.000
**BPV parameters**						
**BHR (per min)**						
Control	90.56 ± 8.09	95.92 ± 10.20	95.93			
Study	91.33 ± 8.11	81.92 ± 6.64	81.91	156.170	< 0.001	0.403
**SBP (mmHg)**						
Control	112.67 ± 8.21	132.70 ± 12.53	132.60			
Study	110.54 ± 8.02	124.83 ± 11.42	125.00	26.265	< 0.001	0.102
**DBP (mmHg)**						
Control	69.88 ± 5.26	78.56 ± 7.47	78.58			
Study	69.40 ± 5.13	71.44 ± 6.29	71.42	63.191	< 0.001	0.215
**MAP (mmHg)**						
Control	84.15 ± 8.71	96.60 ± 7.12	96.59			
Study	83.11 ± 4.36	89.24 ± 6.37	89.25	68.446	< 0.001	0.229
**RPP (mmHg)**						
Control	102.05 ± 10.03	127.17 ± 17.14	127.10			
Study	101.02 ± 12.17	102.27 ± 12.58	102.30	160.439	< 0.001	0.410
**SV (mL)**						
Control	61.28 ± 9.12	65.70 ± 12.17	65.37			
Study	60.26 ± 10.00	60.84 ± 10.86	61.15	10.573	0.001	0.044
**LVET (ms)**						
Control	308.01 ± 22.72	314.60 ± 24.62	314.40			
Study	307.06 ± 21.38	299.98 ± 18.76	300.10	28.129	< 0.001	0.109
**CO (L/min)**						
Control	5.60 ± 1.72	6.27 ± 1.95	6.24			
Study	5.41 ± 1.62	4.91 ± 1.47	4.95	37.159	< 0.001	0.139
**TPR (mmHg min/L)**						
Control	0.99 ± 0.21	1.12 ± 0.31	1.12			
Study	1.04 ± 0.27	0.92 ± 0.23	0.92	30.198	< 0.001	0.116
**BRS (ms/mmHg)**						
Control	12.70 ± 4.17	10.14 ± 3.61	10.14			
Study	13.01 ± 4.75	15.65 ± 5.54	15.64	79.21	< 0.001	0.255
**HRV parameters**						
**SDNN (ms)**						
Control	33.65 ± 12.97	25.29 ± 7.93	25.28			
Study	34.28 ± 13.59	42.18 ± 16.43	42.19	97.929	< 0.001	0.298
**RMSSD (ms)**						
Control	27.86 ± 12.37	21.63 ± 7.57	21.48			
Study	26.17 ± 10.88	33.82 ± 11.18	33.96	100.898	< 0.001	0.306
NN50*						
Control	20.00 (10.00–32.25)	19.00 (13.00–26.50)	19.75			
Study	23.50 (10.00–33.25)	28.00 (18.00–41.00)	29.15	35.374	< 0.001	0.133
pNN50* (%)						
Control	4.90 (3.00–7.12)	4.00 (3.00–5.30)	4.06			
Study	4.80 (2.95–7.00)	6.00 (4.00–8.70)	6.43	45.801	< 0.001	0.165
**TP (ms**^**2**^**)**						
Control	922.42 ± 276.51	713.33 ± 214.98	713.20			
Study	905.23 ± 269.58	1066.23 ± 335.12	1066.00	90.172	< 0.001	0.281
**LFnu**						
Control	54.37 ± 15.62	61.88 ± 17.04	61.90			
Study	55.26 ± 15.71	45.71 ± 15.21	45.68	59.058	< 0.001	0.204
**HFnu**						
Control	45.62 ± 14.80	38.12 ± 12.45	38.06			
Study	44.74 ± 14.81	54.31 ± 18.70	54.37	61.569	< 0.001	0.210
**LF-HF ratio**						
Control	1.21 ± 0.44	1.84 ± 0.87	1.85			
Study	1.44 ± 0.74	1.02 ± 0.66	1.02	64.776	< 0.001	0.219

Pre-Test: recordings before intervention, at 16th week; Post-Test: recordings after 20 weeks of intervention, at 36th week.

Comparison of data between the Control (Post) and Study (Post) was done by analysis of covariance (ANCOVA). The p value < 0.05 was statistically considered significant. * Data presented are median (inter-quartile range) and adjusted median.

BMI body mass index, BHR basal heart rate, SBP systolic blood pressure, DBP diastolic blood pressure, MAP mean arterial pressure, RPP rate pressure product, SV stroke volume, LVET left ventricular ejection time, CO cardiac output, TPR total peripheral resistance, BRS baroreflex sensitivity, SDNN standard deviation of the averages of NN intervals, RMSSD square root of the mean of the sum of the squares of differences between adjacent NN intervals, NN50 number of interval differences of successive NN intervals greater than 50 ms, pNN50 proportion derived by dividing NN50 by the total number of NN interval, TP total power of HRV, LFnu normalized low frequency component, HFnu normalized high frequency component, LF-HF ratio ratio of LF to HF.

Among the HRV indices, there was significant increase in TP (Fig. 16D), SDNN, RMSSD, NN50, pNN50 and HFnu, and significant decrease in LFnu and LF-HF ratio (Fig. 16B) in study group compared to control group after 20-weeks of yoga therapy. The condition effect was maximum for TP (F = 90.172, *p* < 0.001, η^2^ = 0.281), SDNN (F = 97.929, *p* < 0.001, η^2^ = 0.298), RMSSD (F = 100.898, *p* < 0.001, η^2^ = 0.306).

### Biochemical (cardiometabolic risk) parameters

At 36th week, there was significant decrease in FBG, insulin, HOMA-IR, HbA1C, lipid parameters, AIP, hsCRP, IL-6 (Fig. 16E) and MDA, and increase in HDL-C and NO (Fig. 16F) in study group subjects (Table 5). The condition effect was maximum for FBG (F = 148.797, *p* < 0.001, η^2^ = 0.392), LDL-C (F = 144.224, *p* < 0.001, η^2^ = 0.384), LDL-C/HDL-C (F = 140.367, *p* < 0.001, η^2^ = 0.378) and NO (F = 134.626, *p* < 0.001, η^2^ = 0.368).

**Table 5:** Descriptive statistics and ANCOVA results of Post-Test (after 20 weeks of intervention) biochemical parameters between the control (pregnant women with risks of GH receiving routine standard antenatal care alone) and study group (pregnant women with risks of GH receiving routine standard antenatal care + Yoga) subjects.

Parameters	Pre-test	Post-test		Condition effect		
	Mean ± SD	Mean ± SD	Adjusted mean	F	P	Partial eta^2^
**Glucose-related parameters**						
**FBG (mg/dl)**						
Control	77.76 ± 6.81	84.81 ± 6.42	84.85			
Study	77.95 ± 6.75	75.12 ± 6.75	75.09	148.797	< 0.001	0.392
**HbA1c (g%)**						
Control	4.83 ± 0.56	5.78 ± 0.65	5.77			
Study	4.85 ± 0.60	4.78 ± 0.55	4.76	50.120	< 0.001	0.180
**Insulin (μIU/ml)**						
Control	14.49 ± 5.68	16.24 ± 7.92	16.22			
Study	14.28 ± 5.44	11.81 ± 5.33	11.83	25.453	< 0.001	0.099
**HOMA-IR**						
Control	2.78 ± 1.17	3.38 ± 1.69	3.38			
Study	2.75 ± 1.10	2.18 ± 1.00	2.19	44.587	< 0.001	0.162
**Lipid profile**						
**TC (mg/dl)**						
Control	234.27 ± 36.40	240.66 ± 38.61	240.70			
Study	234.55 ± 36.58	216.41 ± 33.75	216.40	26.133	< 0.001	0.102
**TG (mg/dl)**						
Control	140.08 ± 43.84	157.27 ± 46.97	157.30			
Study	141.14 ± 45.18	136.71 ± 40.95	136.70	12.725	< 0.001	0.052
**HDL-C (mg/dl)**						
Control	56.87 ± 12.57	49.32 ± 7.91	49.28			
Study	55.36 ± 12.29	59.64 ± 14.69	59.67	44.127	< 0.001	0.160
**LDL-C (mg/dl)**						
Control	97.43 ± 16.47	105.06 ± 18.49	104.90			
Study	95.40 ± 15.37	80.41 ± 12.28	80.54	144.224	< 0.001	0.384
**VLDL-C (mg/dl)**						
Control	28.02 ± 8.63	31.45 ± 9.39	31.45			
Study	28.23 ± 8.52	27.34 ± 8.19	27.34	12.725	< 0.001	0.052
**Lipid risk factors**						
**TC/HDL-C**						
Control	4.33 ± 1.20	5.01 ± 1.14	5.01			
Study	4.39 ± 0.99	3.85 ± 1.10	3.85	62.097	< 0.001	0.212
**LDL-C/HDL-C**						
Control	1.79 ± 0.51	2.16 ± 0.47	2.16			
Study	1.80 ± 0.46	1.44 ± 0.45	1.44	140.367	< 0.001	0.378
**TG/HDL-C**						
Control	2.60 ± 1.03	3.24 ± 1.04	3.25			
Study	2.69 ± 1.16	2.47 ± 1.12	2.46	30.052	< 0.001	0.115
**AIP**						
Control	0.38 ± 0.17	0.48 ± 0.15	0.48			
Study	0.39 ± 0.16	0.35 ± 0.19	0.35	30.897	< 0.001	0.134
**Inflammatory markers**						
**hsCRP (µM/L)**						
Control	6.92 ± 2.99	9.55 ± 4.53	9.54			
Study	7.25 ± 4.89	5.97 ± 3.65	5.97	43.910	< 0.001	0.160
**IL-6 (pg/ml)**						
Control	8.28 ± 3.53	10.77 ± 5.59	10.79			
Study	8.83 ± 5.09	7.12 ± 4.19	7.11	32.389	< 0.001	0.123
**OS marker**						
**MDA (μM/L)**						
Control	9.35 ± 3.15	11.74 ± 4.95	11.73			
Study	10.40 ± 5.38	9.32 ± 3.95	9.33	16.645	< 0.001	0.067
**ED marker**						
**NO (µM/L)**						
Control	37.19 ± 8.71	27.53 ± 6.78	27.36			
Study	35.23 ± 7.16	43.50 ± 13.38	43.66	134.626	< 0.001	0.368

Pre-Test: recordings before intervention, at 16th week; Post-Test: recordings after 20 weeks of intervention, at 36th week.

Comparison of data between the Control (Post) and Study (Post) was done by analysis of covariance (ANCOVA). The p value < 0.05 was statistically considered significant.

FBG fasting blood glucose, HOMA-IR homeostatic model assessment-insulin resistance, TC total cholesterol, TG triglycerides, HDL-C high density lipoprotein cholesterol, LDL-C low density lipoprotein cholesterol, VLDL-C very low-density lipoprotein cholesterol, AIP atherogenic index of plasma, hsCRP high-sensitive C-reactive protein, IL-6 interleukin-6, OS oxidative stress, MDA malondialdehyde, ED endothelial dysfunction; NO, nitric oxide.

### Correlation and regression analysis of NO with various parameters

**Figure 17:**
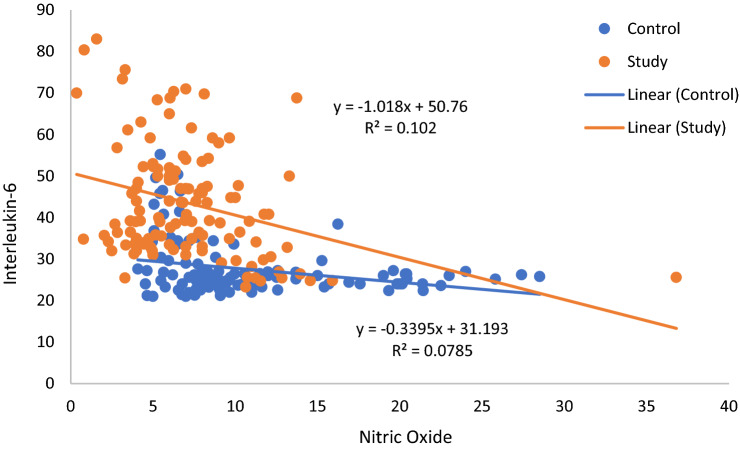
Pearson correlation of nitric oxide with IL-6 in control group and study group at 36th week of gestation. Blue dots represent control group and orange dots represent study group. NO is negatively correlated with IL-6 in both control and study groups and the correlation is stronger in study group (*r* = − 0.319, *p* < 0.001).

At 36th week, all parameters in control group except RPP, SDNN, body weight, duration of labor and labor pain (NLPS) and all parameters in study group except RPP, SDNN, HOMA-IR, AIP, duration of labor and labor pain (NLPS) were significantly correlated with nitric oxide (Table 6). The correlation of decreased IL-6 with increased NO had maximum significance in study group following 20 weeks of yoga intervention (Fig. 17).

**Table 6:** Pearson correlation of nitric oxide with various parameters in women having risk factor for gestational hypertension in control group (n = 113) and study group (n = 121) at 36th week of gestation.

Parameters	Control group		Study group	
	At 36th week		At 36th week	
	*r*	P	*r*	P
SBP	− 0.194	0.040	− 0.187	0.040
DBP	− 0.231	0.014	− 0.188	0.039
MAP	− 0.304	0.001	− 0.276	0.002
RPP	− 0.087	0.359	0.025	0.787
BRS	0.199	0.035	0.255	0.005
TP	0.225	0.017	0.323	<0.001
LF-HF ratio	− 0.208	0.027	− 0.201	0.027
SDNN	0.153	0.106	0.105	0.250
RMSSD	0.353	< 0.001	0.191	0.036
HOMA-IR	− 0.199	0.035	− 0.073	0.423
AIP	− 0.227	0.016	0.037	0.690
MDA	− 0.268	0.004	− 0.222	0.014
hsCRP	− 0.183	0.052	− 0.190	0.036
IL-6	− 0.278	0.003	− 0.319	<0.001
Birth weight	0.013	0.891	0.240	0.008
Duration of labor	− 0.028	0.769	− 0.174	0.054
Labor pain (NLPS)	0.051	0.591	− 0.050	0.584

The P value < 0.05 was considered significant, *r* correlation coefficient.

SBP systolic blood pressure, DBP diastolic blood pressure, MAP mean arterial pressure, RPP rate pressure product, BRS baroreflex sensitivity, TP total power of HRV, LF-HF ratio of LF to HF, SDNN standard deviation of normal to normal (NN) interval, RMSSD square root of the mean of the sum of the squares of the differences between adjacent NN intervals, HOMA-IR homeostatic model assessment-Insulin resistance, AIP atherogenic index of plasma, MDA malondialdehyde, hsCRP high sensitive C-reactive protein, IL-6 interleukin-6, NPLS numeric labor pain scale.

In multiple regression analysis of control group, MAP (β =  − 0.218, *p* = 0.002), BRS (β = 0.432, *p* < 0.001), TP (β = 0.184, *p* = 0.016) and IL-6 (β =  − 0.150, *p* = 0.034) had significant independent contribution to the nitric oxide at 36th week (Table 7). In the study group, MAP (β =  − 0.258, *p* = 0.002), BRS (β = 0.305, *p* < 0.001), TP (β = 0.187, *p* = 0.024), IL-6 (β =  − 0.194, *p* = 0.022), and birth weight (β = 0.163, *p* = 0.038), had significant independent contribution to the nitric oxide at 36th week (Table 8).

**Table 7:** Multiple regression analysis of nitric oxide (as dependent variable) with various parameters (as independent variables) in control group at 36th week of gestation.

Independent variables	Standardized regression coefficient β	95% CI		P values
		Lower limit	Upper limit	
MAP	− 0.218	− 0.339	− 0.077	0.002
BRS	0.432	0.514	1.105	< 0.001
TP	0.184	0.001	0.011	0.016
HOMA-IR	− 0.043	− 0.787	0.446	0.584
IL-6	− 0.150	− 0.349	− 0.014	0.034
MDA	− 0.056	− 0.282	0.128	0.456
AIP	0.086	− 2.622	3.371	0.240

C.I. confidence interval, MAP mean arterial blood pressure, BRS baroreflex sensitivity, TP total power of HRV, HOMA-IR homeostatic model assessment-Insulin resistance, IL-6 interleukin-6, MDA malondialdehyde, AIP atherogenic index of plasma.

**Table 8:** Multiple regression analysis of nitric oxide (as dependent variable) with various parameters (as independent variables) in study group at 36th week of gestation.

Independent variables	Standardized regression coefficient β	95% CI		P values
		Lower limit	Upper limit	
MAP	− 0.258	− 0.931	− 0.210	0.002
BRS	0.305	0.132	1.120	< 0.001
TP	0.187	0.001	0.314	0.024
IL-6	− 0.194	− 1.148	− 0.089	0.022
MDA	− 0.125	− 0.993	0.144	0.142
BW	0.163	− 1.757	0.336	0.038

C.I. confidence interval, MAP mean arterial pressure, BRS baroreflex sensitivity, TP total power of HRV, IL-6 interleukin-6, MDA malondialdehyde, BW birth weight.

## Discussion

In the present study, the incidence of GH was 25.66% in women having risk of PIH who did not practice yoga (control group), whereas the incidence was only 6.61% in the study group subjects who had practiced twenty weeks of yoga (Table 3). Also, there was no incidence of preeclampsia or eclampsia in the study group. Overall, 38.1% subjects in the control group developed HDP, whereas 6.61% subjects had HDP in study group. Further, the risk of developing GH was significantly reduced (RR 2.65; CI 1.42–4.95; *p* < 0.001) in study group following yoga intervention. Thus, finding of the present study indicate that practice of twenty weeks yoga during second and third trimesters of pregnancy could significantly reduce the risk of developing hypertension during pregnancy. There was significant decrease (*p* < 0.001) in SBP, DBP and MAP (Fig. 16A) in study group (Table 4). In control group, 43 subjects had SBP 140 mmHg or more and 14 among these 43 subjects had DBP 90 mmHg or above, whereas in study group 8 subjects had SBP 140 mmHg or more and none of them had DBP 90 mmHg or above (Table 1). These data indicate that the practice of yoga led to the apparent differences in incidence of diagnosis of HDP. However, all patients in both the groups who met the criteria of HDP were placed on antihypertensive medications. Therefore, it can’t be concluded that yoga alone was effective in reducing BP in these patients. Though there is a recent report of effects of yoga reducing systolic and diastolic blood pressures in hypertensive disorder of pregnancy^11^, the duration of practice of yoga was four weeks, the sample size was 30, only primigravida after developing hypertension were recruited in the study, all forms of pregnancy-hypertension (GH, preeclampsia and eclampsia) were included in the study and no mechanistic aspects of yoga were addressed. There are also reports of effectiveness of yoga in HDP^9^, but subjects in these studies were pregnant women who had already developed hypertension. To best of our knowledge, there is no report of effects of yoga practiced for 20 weeks and followed-up till delivery in women having risk of HDP. As GH is the new-onset hypertension that occurs after 20th week of gestation, in the present study yoga was instituted at 16th week of pregnancy. However, yoga could not be instituted prior to 16th week of gestation, as in our hospital the pregnant women come for antenatal check-up usually after 1st trimester of pregnancy.

A major part of the yoga schedule in the present study was the basic asanas that are reported to reduce sympathetic tone and stress in risk pregnancies^10,26^, and the pranayamas that are known to enhance the vagal tone in hypertensive pregnancies^11,12^. The four pranayamas adopted in the yoga module of the study were slow breathing techniques that are well recognised to improve vagal tone, HRV and CV functions in health and disease^10–12^. There was substantial decrease in heart rate (BHR) and improvement in heart rate variability (increase in TP of HRV) in study group compared to control group (Table 4, Fig. 16D), which indicates a significant increase in vagal drive of cardiac regulation following twenty weeks of yoga practice, as TP is an index of overall cardiac vagal modulation^24,30^. Time-domain indices of HRV (SDNN, RMSSD, NN50 and pNN50), the markers of cardiac vagal drive^24^, were increased prominently in yoga group, which was associated with decreased LF-HF ratio (Fig. 16B). Decrease in LF-HF ratio indicates improved sympathovagal balance with decreased sympathetic drive^24,30^. These findings denote substantial improvement in HRV, increased vagal control of cardiac modulation, and decreased sympathetic drive after twenty weeks of yoga practice. Decreased heart rate and increased TP of HRV are associated with decreased CV events and all-cause mortality^30^. The RPP, an important marker of myocardial work stress^31^, was prominently decreased in study group compared to control group and RPP had the highest condition effect (F = 160.439, *p* < 0.001, η^2^ = 0.410) among all the CV parameters (Table 4). Increased heart rate and RPP are known markers of CV risks, especially in hypertensive subjects^31^. Thus, decrease in heart rate and RPP in the study group subjects after twenty weeks yoga intervention indicates decreased CV risk in these subjects. Further, considerable increase in BRS after yoga intervention (F = 79.217, *p* < 0.001, η^2^ = 0.255) (Table 4, Fig. 16C) signifies decreased CV risk following twenty weeks of yoga practice, as decreased BRS is a predictive CV risk marker in PIH^17,18^.

It has been proposed that practice of slow pranayamic breathings such as anulom-vilom, bhramari and sheetali increases blood NO level that in-turn decreases BP and reduces CV risks^12,20^. Recently, we have reported that sympathovagal imbalance and CV risks are linked to decreased NO in women having risks of GH^18^. In the present study, significantly increased NO level in study group subjects following yoga intervention (Fig. 16F) had substantial condition effect (F = 134.626, *p* < 0.001, η^2^ = 0.368) among all the biochemical parameters (Table 5). Decrease in NO was significantly correlated with increase in MAP and decrease in BRS in control group and increase in NO was significantly correlated with decrease in MAP and increase in BRS in study group (Table 6). NO had maximum correlation with BRS (r = 255, *p* = 0.005) in study group subjects. Further, increased MAP and decreased BRS and TP had independent association with decreased NO in control group (Table 7) and decreased MAP and increased BRS and TP had independent association with increased NO in study group (Table 8), as demonstrated by multiple regression analysis. These findings signify that decrease in BP and CV risks in women with risk of GH following practice of yoga for twenty weeks is linked to increase in the level of NO in these subjects.

Decrease in fasting blood glucose following twenty weeks of yoga practice in study group had maximum condition effect (F = 148.797, *p* < 0.001, η^2^ = 0.392) (Table 5). Also, there was significant decrease in HbA1C in the study group following practice of yoga (F = 50.120, *p* < 0.001, η^2^ = 0.180) indicating improvement in glycemic control in yoga treated subjects. However, HOMA-IR was not significantly correlated with NO in study group, which indicates that though practice of yoga decreases insulin resistance, it is not linked to the increase in NO level in these women. Though the lipid profile of subjects of the present study was not compared with that of a nonpregnant group, the lipid profile appears to be atherogenic as compared to the lipid profile data we have reported earlier in normal pregnant women^32^. Belo et al. have reported that human gestation is associated with an atherogenic lipid profile, which is further enhanced in preeclampsia^33^. Although, the lipid profile and lipid risk factors were decreased following yoga intervention (Table 5), the decrease in atherogenic index was not correlated with NO (Table 6). Also, oxidative stress (levels of MDA) was significantly decreased in study group following intervention, but was not linked to NO, as MDA had no independent contribution to NO in both groups, demonstrated by regression models (Tables 7 and 8).

There was substantial decrease in the level of hsCRP and IL-6 (Fig. 16E), the markers of inflammation in study group subjects (Table 5) and as revealed by multiple regression analysis, the decreased level of IL-6 had independent association with increased NO (β = − 0.194, *p* = 0.022) following twenty weeks of yoga intervention (Fig. 17). These findings illustrate that decreased inflammatory milieu following practice of yoga in women with risk of GH is linked to the increase in NO, the marker of endothelial dysfunction^18^. Inflammation as a potential contributor to genesis of GH^15^ and the role of IL-6 in causation of hypertension during pregnancy are well documented^15,16^. Also, practice of yoga has been reported to decrease inflammatory biomarkers including IL-6^21^. There is a recent report of practice of bhramari pranayama enhancing the release of nitric oxide that prevents coagulopathies and morbidity in Coronavirus Disease (COVID-19)^34^. Thus, increased plasma NO level by deep and slow breathing pranayamas in the present study could have prevented the development of hypertension and cardiometabolic risks and decreased the inflammatory milieu in pregnant women having risk of GH.

There was no premature delivery, no PROM, considerably less IUGR (0.82%), more number of spontaneous vaginal delivery (SVD) (96.7%), and less pain during delivery (97.1%), in the study group subjects compared to the control group subjects (Table 3). The birth weight of neonates was significantly high in study group (*p* = 0.001) and there was a significant correlation (r = 0.240, *p* = 0.008) (Table 6), and independent association of birth weight with NO (β = 0.163, *p* = 0.038) in the study group (Table 8). The duration of labor was close to significance in study group (r = − 0.174, *p* = 0.054). These findings indicate that maternal, fetal and neonatal outcomes are considerably improved following twenty weeks yoga practice in study group subjects and decreased maternal BP and increased neonatal birth weight could be linked to the increased level of NO. This is the first report of an RCT of twenty weeks practice of a structured yoga programme on fetomaternal-neonatal outcomes in pregnant women having risk of GH. Recently we have reported improvement of fetomaternal outcomes and CV health following a short-course yoga therapy consisting mainly of slow pranayamas and basic relaxing asanas in women having risk of developing gestational diabetes mellitus^35^. Though there are earlier reports of improvement in pregnancy outcomes by yoga in HDP^9–11^, the design and data analysis were different and cause-effect relationships have not been assessed in those studies.

The merits of the present study comprise this is an RCT conducted in women with risk of developing GH recruited from the early part of 2nd trimester before they developed hypertension, yoga module consisting of asanas and pranayamas that are known to improve pregnancy outcomes was included in the schedule, yoga intervention was imparted for twenty weeks with close monitoring of the compliance of practice, entire gamut of CV risk parameters including HRV and BRS was part of the assessments, contribution of cardiometabolic factors to the endothelial dysfunction was assessed, and appropriate methods of data analysis in an RCT were followed. However, the major limitation of the study is that we did not recruit the subjects in their early prenatal visits in the first trimester and we have not substantiated the influence of earlier enrolment and intervention on the outcomes. We have also not assessed the circulating soluble fms-like tyrosine kinase-1 (sFlt-1) and placental growth factor (PlGF), the diagnostic markers of preeclampsia and we have not measured the other important vascular endothelial dysfunction markers such as vascular endothelial growth factor (VEGF). Nevertheless, the present study is the novel one in demonstrating the safety efficacy of a doable yoga schedule in preventing the development of hypertension and improving the fetomaternal outcomes in pregnant women having risk of GH. As such prevalence of GH in South Asian nations is quite high^2,3^, and healthcare delivery systems are not well developed in these counties. Therefore, early intervention of yoga in pregnant women having risk of GH, which is cost-effective and apparently safe in pregnancy, will prevent the development of GH and will improve the fetomaternal and neonatal outcomes in these high-risk mothers.

## Summary

The present RCT was conducted in women with risk of developing GH recruited from the early part of 2nd trimester before they developed hypertension. The yoga module consisting of asanas and pranayamas that are known to improve pregnancy outcomes was included in the schedule and yoga intervention was imparted for twenty weeks with close monitoring of the compliance of yoga practice. The CV risk parameters including HRV and BRS were measured and the contribution of cardiometabolic factors to the endothelial dysfunction was assessed. Twenty-week yoga practice decreased the incidence of hypertension, improved the fetomaternal-neonatal outcomes and reduced the cardiometabolic risks in pregnant women having risk of GH. Decreased BP, increased HRV, BRS and birth weight and decreased inflammation were linked to improved endothelial function. Thus, findings of the present study highlight the potential benefits of integration of yoga practice in the management of risk pregnancies, especially for women having risk of GH. It is proposed that yoga therapy module of the present study should be part of the medical management in the treatment of hypertensive disorders of pregnancy. Also, it is suggested that yoga should be instituted in the early part of pregnancy, especially in high-risk pregnant women for a healthy pregnancy and comfortable delivery.

## Data Availability

The datasets used and/or analysed during the current study are available from the corresponding author on reasonable request.

## Abbreviations

GH: Gestational hypertension
BRS: Baroreflex sensitivity
HRV: Heart rate variability
VED: Vascular endothelial dysfunction
NO: Nitric oxide
HDP: Hypertensive disorders of pregnancy
BP: Blood pressure
ASA: Acetylsalicylic acid
PIH: Pregnancy induced hypertension
CV: Cardiovascular
BMI: Body mass index
BHR: Basal heart rate
SBP: Systolic blood pressure
DBP: Diastolic blood pressure
TP: Total power
LFnu: Low frequency power expressed in normalized units
HFnu: High frequency power expressed in normalized units
LF-HF ratio: Ratio of low-frequency to high-frequency power
SDNN: Mean and standard deviation of RR intervals
RMSSD: Square root of the mean of the sum of the squares of differences between adjacent RR interval
NN50: Adjacent RR interval differing more than 50 ms
pNN50: NN50 counts divided by all RR intervals
BPV: Blood pressure variability
MAP: Mean arterial pressure
RPP: Rate-pressure product
LVET: Left ventricular ejection time
SV: Stroke volume
TPR: Total peripheral resistance
HOMA-IR: Homeostatic model assessment of insulin resistance
TC: Total cholesterol
TG: Triglyceride
HDL-c: High-density lipoprotein-cholesterol
LDL-c: Low-density lipoprotein-cholesterol
VLDL-c: Very low density lipoprotein-cholesterol
AIP: Atherogenic index of plasma
MDA: Malondialdehyde
hsCRP: High sensitive C-reactive protein
IL-6: Interleukin-6
IUGR: Intrauterine growth retardation
PROM: Premature rupture of membrane
